# Structural and Kinetic Basis for the Rational Design of Next-Generation β‑Lactamase Inhibitors

**DOI:** 10.1021/acs.jmedchem.5c03315

**Published:** 2026-03-16

**Authors:** Shuang Chen, Muchen Yu, Manming Xu, Sergio Decherchi, Magdalena A. Taracila, Andrea M. Hujer, Christopher R. Bethel, Robert A. Bonomo, Shozeb Haider

**Affiliations:** † Department of Pharmaceutical and Biological Chemistry, School of Pharmacy, 4919 University College London, London WC1N 1AX, U.K.; ‡ Data Science and Computation Facility, Fondazione Istituto Italiano di Technologia, Via Morego 30, Genoa 16163, Italy; § Department of Molecular Biology and Microbiology, Case Western Reserve University School of Medicine, Cleveland, Ohio 44106-5029, United States; ∥ Department of Medicine, 12304 Case Western Reserve University School of Medicine, Cleveland, Ohio 44106-5029, United States; ⊥ Clinician Scientist Investigator, Louis Stokes Cleveland Department of Veterans Affairs Medical Center, Cleveland, Ohio 44106-1702, United States; # Departments of Pharmacology, 2546 Biochemistry, and Proteomics and Bioinformatics, Case Western Reserve University School of Medicine, Cleveland, Ohio 44106-5029, United States; ¶ CWRU-Cleveland VAMC Center for Antimicrobial Resistance and Epidemiology (Case VA CARES), Cleveland, Ohio 44106-5029, United States; ∇ Prince Fahd Bin Sultan Chair for Biomedical Research (PFSCBR), University of Tabuk, Tabuk 71491, Saudi Arabia

## Abstract

The global spread of β-lactamase-mediated resistance poses a threat to β-lactam antibiotics. Boron-based β-lactamase inhibitors (BLIs) represent a promising class of reversible covalent inhibitors, yet the molecular basis of their recognition and dissociation remains poorly understood. Using *Pseudomonas*-derived cephalosporinase-3 (PDC-3) as a model, we employed enhanced sampling strategies with machine learning and steady-state kinetic assays to investigate the binding and unbinding dynamics of LP06, a boronate BLI. We identify three binding pathways, governed by hydrophobic recognition motifs and a conserved arginine anchor that together steer the ligand toward the precovalent state. Sequence alignment of nearly 7000 class C β-lactamases supports the conservation of these determinants, and structural analyses suggest that R349 may act as a shared anchoring point across serine β-lactamases. Additionally, hydrogen-bonding interactions were found to delay productive binding by stabilizing nonproductive conformations. Our findings provide fundamental insights into β-lactamase inhibition and establish design principles for next-generation β-lactamase inhibitors.

## Graphical abstract

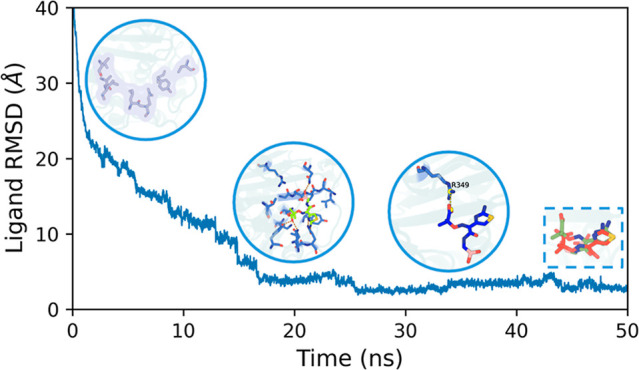

## Introduction

β-Lactam antibiotics, including penicillins, cephalosporins, carbapenems, and monobactams, have long been effective against bacterial infections.^1^ However, the emergence of antimicrobial resistance, especially through the production of β-lactamase enzymes by bacteria, poses a significant threat to their efficacy.^2^ In the Ambler classification system, β-lactamases are classified into A, B, C, and D according to their amino acid sequence.^3^ Among these enzymes, class C β-lactamase, also known as AmpC or cephalosporinase, is prevalent in Gram-negative bacteria such as *Pseudomonas aeruginosa*.^4^ According to the Beta-Lactamase Database (BLDB; http://www.bldb.eu/), class C β-lactamases represent nearly 7000 entries out of 12,000 documented β-lactamases (∼58.3%; Supporting Information 1).^5^ Class C β-lactamases exert their resistance mechanisms, primarily through the hydrolysis of cephalosporins mediated by a structurally conserved active site. The active site comprises two distinct regions: the R1 site, bound by the Ω-loop, and the R2 site, encased by the R2-loop. These regions interact with the R1 and R2 side chains of cephalosporins, respectively (Figure 1).^6^

**Figure 1:**
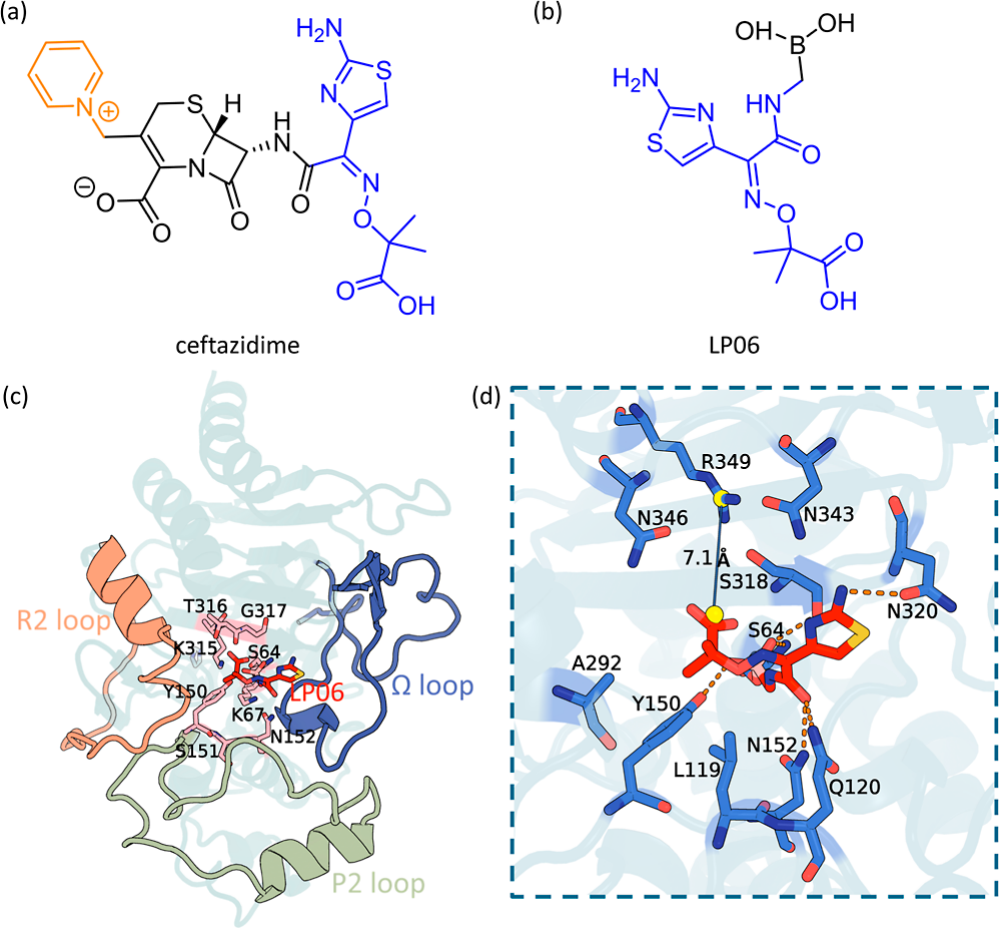
Structures of ceftazidime and LP06, pseudomonas-derived cephalosporinase 3 (PDC-3) and interactions of the PDC-3–LP06 complex. (a) Chemical structure of ceftazidime, with the R1 (blue) and R2 (orange) side chains highlighted. (b) Chemical structure of LP06, with the region shared with ceftazidime colored in blue. (c) Structural representation of the crystal structure of PDC-3 bound to LP06 (PDB ID: 8SDR). The key regions of the enzyme, including the Ω loop (purple), R2 loop (orange), and P2 loop (green), are highlighted. The residues in the conserved catalytic motifs are shown as pink sticks. (d) A close-up of the binding site interactions between LP06 and PDC-3. The hydrogen bonds are depicted as orange dashed lines, and the distance between R349 and the carboxylate group of LP06 is shown as a blue solid line.

To counteract resistance caused by β-lactamases, a common method is the synergistic use of β-lactamase inhibitors (BLIs) with β-lactams.^7^ Boron-based BLIs are transition-state analogues and are among the most promising non-β-lactam BLIs. They function by rapidly forming a reversible covalent bond with the active site serine through a tetrahedral intermediate, similar to those formed during β-lactam ring hydrolysis.^8^ Vaborbactam was the first FDA-approved BLI.^9,10^ Vaborbactam is paired with the carbapenem antibiotic Meropenem for treating complicated urinary tract infections and other serious infections caused by resistant Gram-negative bacteria.^9,10^ Subsequently, taniborbactam, a bicyclic boronate inhibitor currently in late-stage development, has completed Phase III clinical trials in combination with cefepime^11^ and has shown enhanced efficacy. Xeruborbactam can also inhibit all four classes of β-lactamases at low concentrations.^12,13^

Recently, preclinical candidates have been developed that merit attention. Two α-amido-β-triazolylethaneboronic acid transition state inhibitors, S02030 and MB076, were evaluated against KPC-2 and CTX-M-96 with nanomolar-range IC_50_ values (2 to 135 nM).^14^ Furthermore, LP06 is a prototypical boronic acid derivative that mimics the structure of ceftazidime R1 side chain, enabling it to effectively inhibit β-lactamases like ADC-7 (*K*_i_ = 50 nM), CTX-M-9 (*K*_i_ = 15 nM), and CTX-M-16 (*K*_i_ = 4 nM), effectively restoring ceftazidime susceptibility.^15,16^ Previous studies have also shown that PDC-3 and its variants, E219K and Y221H, are all inhibited by LP06 with nanomolar IC_50_ values.^17^

However, the vast diversity and remarkable adaptability of β-lactamases, particularly the extensive class C family, require continual improvement in inhibitor design. A deeper understanding of these dynamic processes is crucial for guiding the design of new inhibitors with enhanced potency and spectrum.^17,18^ However, experimental capture of these processes is nearly impossible due to the inherent limitations of current laboratory techniques in resolving such transient and dynamic events.^19,20^ Furthermore, these processes occur on microsecond to millisecond time scales, making them highly challenging for conventional molecular dynamics (MD) simulations, which require significant computational resources to access such time scales.^21,22^ To address these limitations, enhanced-sampling methods such as metadynamics (MetaD),^22^ ligand Gaussian accelerated molecular dynamics (LiGaMD),^23^ adaptive biasing force,^24^ parallel cascade selection molecular dynamics (PaCS-MD),^25^ supervised molecular dynamics,^26,27^ MD-Binding (MDBind),^28^ and scaled molecular dynamics (SMD)^29^ have been developed.

LP06 is one of the most extensively studied preclinical boron-based BLI, and many LP06−β-lactamase complex structures have been deposited in the Protein Data Bank (Supporting Information Note 1). We therefore selected LP06 as a representative preclinical boron-based inhibitor to elucidate general principles governing inhibitor binding to and unbinding from β-lactamases. In this work, we focus on the most predominant β-lactamase class and use PDC-3 as a representative model system. PDC-3 is one of the common variants of the wild-type AmpC of *P. aeruginosa* PAO1 (PDC-1) with a T79A substitution.^30^ (All residues in this study are numbered on the Structural alignment-based numbering of class C β-lactamase scheme.^31^) This substitution enhances the enzyme’s ability to hydrolyze ceftazidime, a third-generation cephalosporin with broad-spectrum activity.^32^ Although the crystal structure of PDC-3 bound to LP06 has provided valuable structural insights, the molecular mechanisms governing LP06 binding to PDC-3, as well as its unbinding pathways, remain unclear.^17,18^ We used the MDBind method to explore the binding pathways of LP06 to PDC-3 and SMD to investigate its unbinding mechanisms. MDBind is an adaptive steered MD method in which ligand and protein binding site atoms attract each other via a Yukawa potential whose charges are not physical but always positive or negative based on the molecular partners.^28^ The method is termed adaptive because the ligand follows electrostatic field lines, and the spring constant is adjusted during steering to render the process as smooth as possible. For the unbinding process, simulations of the protein–ligand complex were carried out under scaled potential energy conditions to lower kinetic barriers and facilitate the unbinding process while simultaneously applying a very weak restraint on the protein backbone to avoid possible unfolding.^29^ To analyze high-dimensional conformational ensembles, we employ a self-organizing map (SOM), which is an unsupervised machine-learning approach. Unlike linear projections (e.g., PCA and tICA), which assume a linear subspace and can merge low-variance yet functionally relevant microstates along curved manifolds, SOM is a nonlinear, topology-preserving mapper that learns prototype nodes from per-frame structural features and clusters them into interpretable neighborhoods, yielding clearer state partitions and pathway visualization.^33,34^ Thus, key residues, critical pathways, and novel kinetic traps were identified, providing an extensive understanding of the behavior of the ligand within class C β-lactamases. Complementary steady-state kinetic assays support the computational predictions. Furthermore, comprehensive multiple sequence alignment of all documented class C β-lactamase sequences, along with structural alignments of representative enzymes from classes A, C, and D, strongly supports the generalizability of the insights presented in this study beyond the specific ligand–enzyme pair analyzed. Hence, our findings offer broadly applicable design principles that can guide the development of next-generation β-lactamase inhibitors with improved binding affinity and prolonged retention.

## Results and Discussion

### Identification of Binding Pathways

Among the 27 trajectories obtained through MDBind simulations, 10 successfully reached the binding site, as indicated by a center-of-mass (COM) distance between the ligand and the binding site below 5 Å (Figure S1). Of the 10 trajectories that achieved a final COM distance under 5 Å, only replicas 4, 6, and 10 adopted orientations and conformations closely resembling the crystal structure of PDC-3 bound to LP06 (Figures 2a, S2, and Supporting Information 2). The three replicas exhibited root-mean-square deviation (RMSD) values of 1.89, 1.89, and 1.98 Å, respectively (RMSD was calculated on the atomic positions of the ligand from the reference crystal structure after aligning the protein’s Cα atoms; Figure S1). Furthermore, only these three trajectories showed the ligand boron atom (B_ligand_) positioned close to the oxygen atom of residue S64 (O_S64_), which is expected to form a covalent bond during the acylation step.^17^

**Figure 2:**
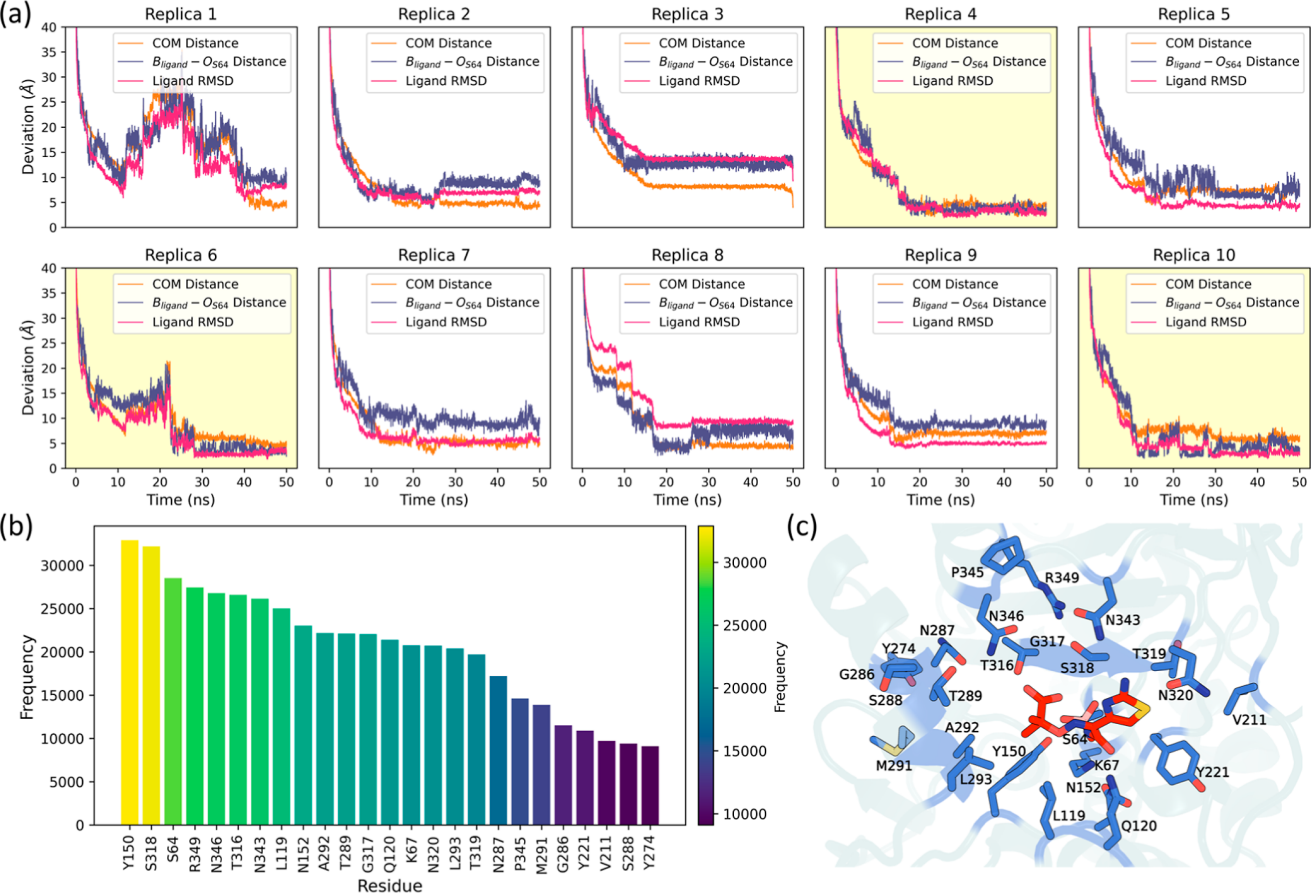
Geometric analysis of binding trajectories. (a) Time evolution of key geometric parameters across 10 replicas of LP06 binding to PDC-3, including the COM distance between the ligand and binding site identified by NanoShaper, B_ligand_–O_S64_ distance, and the RMSD of ligand heavy atoms (upon optimal alignment of protein Cα atoms). Yellow highlights indicate replicas that achieved conformations closely resembling the crystal structure. (b) Frequency of the top 25 residues most frequently in contact (within 4.5 Å) with the ligand across all binding trajectories, ranked from highest to lowest. Residues with higher frequencies are color-coded from yellow (high) to purple (low), highlighting the critical residues involved in ligand binding. (c) Structural mapping of the top 25 contact residues onto the PDC-3 enzyme, depicted in stick representation.

To gain deeper insights into the binding process, an unsupervised machine learning method, an SOM was used to cluster and analyze the trajectory data.^33,34^ The SOM method facilitates the clustering and representation of high-dimensional conformational data in a low-dimensional space, enabling the identification of key intermediate states and transition pathways.^35–37^ The distance between the heavy atoms of the ligand and the heavy atoms of the 25 residues most frequently in contact with the ligand (within 4.5 Å) across all binding trajectories was used as input for the SOM analysis (Figures 2b,c, S3, and S4). The resulting SOM classified the conformational states into 14 distinct clusters (Figure 3a,b). The ligand RMSD relative to the crystal structure (PDB ID: 8SDR) was used as a validation metric for SOM clustering performance. Most of the clusters are characterized by relatively consistent conformations, as indicated by narrow ligand RMSD (aligned to protein Cα atoms) distributions (Figure S5a). However, clusters B–I exhibit broad RMSD distributions. The broader ligand RMSD distributions do not indicate poor SOM clustering. Instead, they reflect the dynamic nature of these states. The time evolution of cluster assignments shows that B, E, H, and I are predominantly sampled at the early stage of the binding process (Figure S6), when the ligand is not yet tightly constrained by the pocket interaction network and remains dominated by weak and transient surface contacts. This results in increased conformational variability, including local fluctuations and small translations. For clusters B, H, and I, structural overlays further indicate that the ligand still occupies the same spatial region with a consistent orientation, and the variability mainly arises from localized adjustments rather than qualitatively different binding locations (Figure S5b–d). In contrast, cluster E encompasses multiple subconformations, including states in which the ligand is far from the protein surface and early stage binding states where the ligand transiently engages with surface residues.

**Figure 3:**
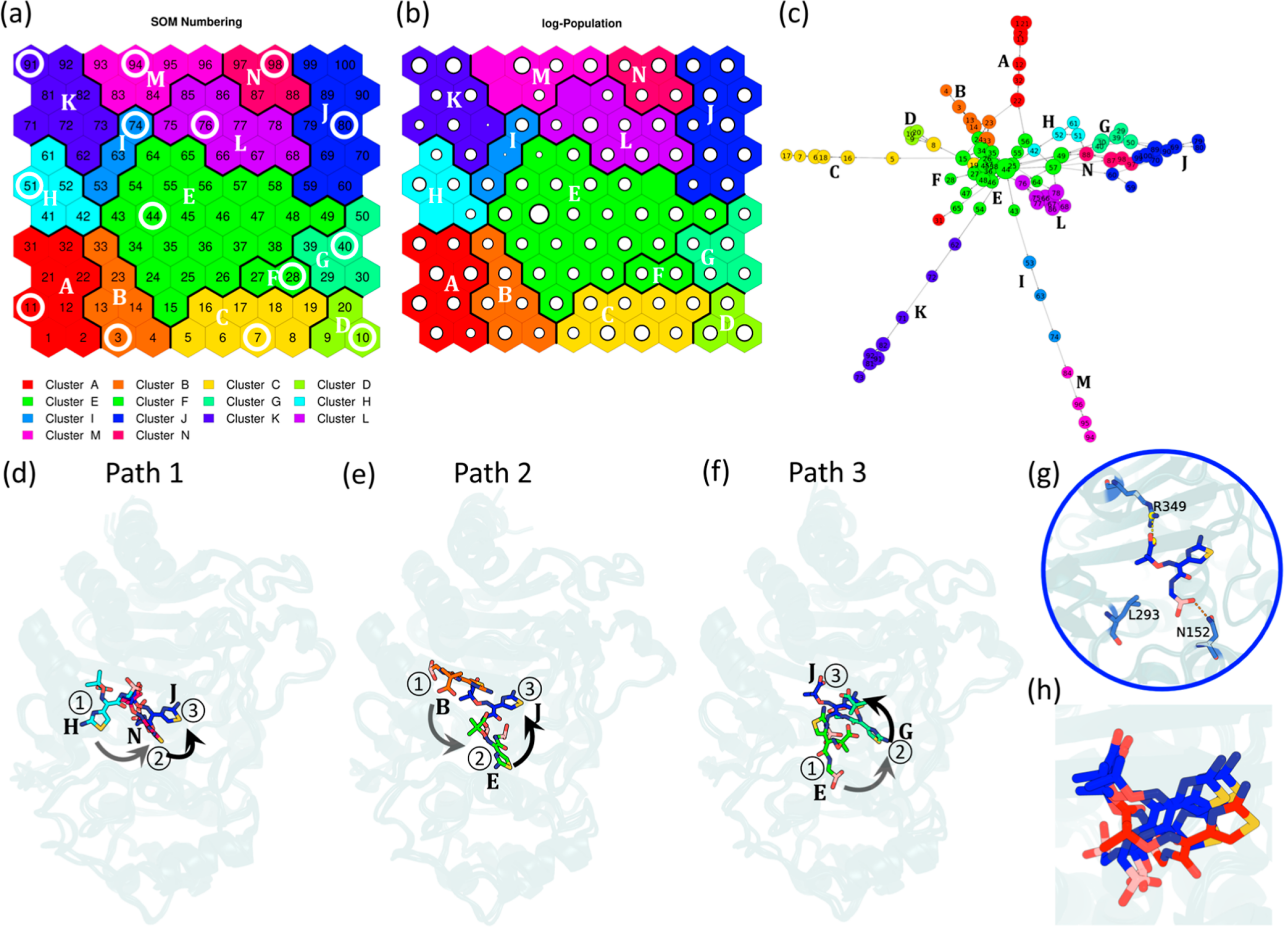
SOM-based analysis of LP06 binding pathways to PDC-3. (a) SOM grid numbering and clustering results. The SOM clusters are color-coded (Cluster A–N), and each hexagon represents an SOM node corresponding to conformational states observed during the simulations. Representative nodes with the highest frame populations are highlighted with white rings. (b) Log-population map of the SOM nodes, where the size of the white circles represents the number of frames assigned to each node. Larger circles indicate higher occupancy. (c) The transition network between SOM nodes, with nodes color-coded based on their clusters (Clusters A–N). (d–f) Structural transitions along the binding pathways for Path 1 (Replica 4, d), Path 2 (Replica 6, e), and Path 3 (Replica 10, f). (g) The representative structure from Cluster J (crystal-like bound state). (h) Representative structures in the final stages of three trajectories (Replica 4, Replica 6, and Replica 10) closely resembling the crystal structure of LP06 bound to PDC-3. The ligand from the crystal structure is shown in red, while the ligands from Replica 4, Replica 6, and Replica 10 are shown in blue.

Through the transition paths of the 10 trajectories, we observed that Cluster B (orange), Cluster E (green), and Cluster H (cyan) correspond to the initial states (Figures S6 and S7), while Cluster J (blue) is predominantly sampled at the final stage of binding and shows the lowest ligand RMSD relative to the LP06–PDC-3 crystal structure among all clusters (Figures S5a,S6 and S7). In addition, three distinct binding pathways were observed, which revealed three modes of initial recognition (Figures 3c–f,4a–f and Supporting Information Movie 1). In Path 1 (H → N → J; Figure 3d), initial recognition occurs near the R2 loop (Figure 4a), where the thiazole group of LP06 engages in hydrophobic interactions with residues such as P290 and M291 (Helix 10), while the boronic acid group forms hydrogen bonds with N346 and R349 (Helix 11). Subsequently, R349 forms a stable salt bridge with the carboxyl group of LP06 (Figure 4d), facilitating its transition to the active site. Similarly, Path 2 (B → E → J; Figure 3e) also highlights the importance of helices 10 and 11 during the early binding stage (Figure 4b), since the ligand is positioned between these helices and the conserved catalytic motif K315TG.^38^ Over time, the ligand gradually shifts toward the P2 loop, where the thiazole group interacts hydrophobically with L119 and Q120, while the dimethyl group engages residues such as T289, A292, and L293 in the R2 loop (Figure 4e). In contrast, Path 3 (E → G → J; Figure 3f) begins with the dimethyl group of LP06 forming hydrophobic interactions with L119 and Q120 in the P2 loop and L293 in the R2 loop (Figure 4c). The ligand then transitions to a position adjacent to the Ω loop, where the thiazole group engages in hydrophobic interactions with Y221 and V211. Simultaneously, the boronic acid group interacts with S64 and S318, which constitute the oxyanion hole, while the carboxyl group establishes hydrogen bonds with N343 (Figure 4f). In the final stage of binding, representative structures from all three trajectories closely resembled the crystal structure of LP06 bound to PDC-3 (Figure 3g,h).

**Figure 4:**
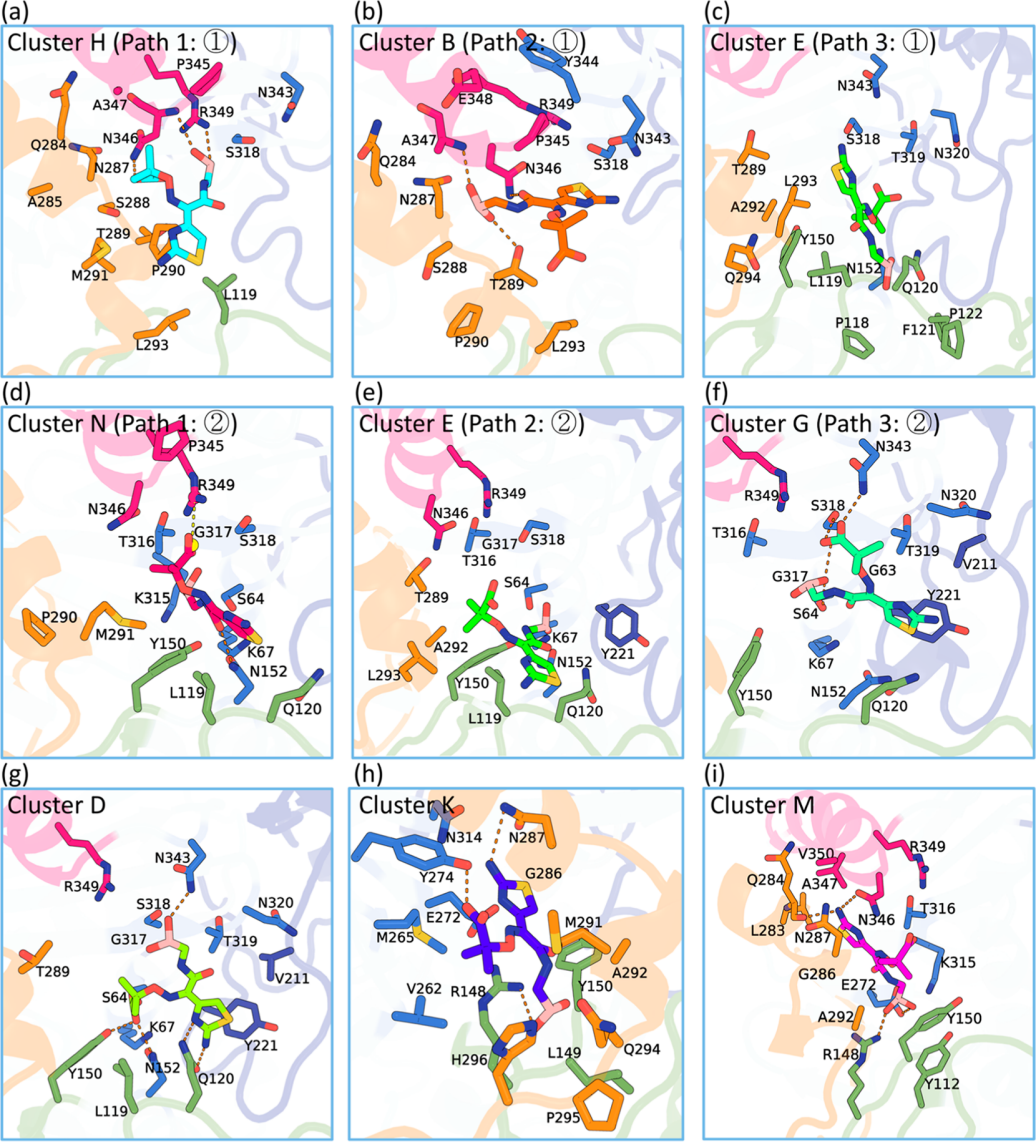
Key conformations along LP06 binding pathways to PDC-3. (a–f) Initial recognition states and transition states observed along the binding pathways. (g–i) Representative structures of kinetic traps from Cluster D, K, and M. The Ω-loop is shown in purple, the R2 loop in orange, the P2 loop in green, and Helix 11 in pink. Salt bridges are shown as orange dashed lines, and hydrogen bonds are shown as yellow dashed lines.

From the crystal structure of PDC-3 bound to LP06, the carboxyl group of LP06 is positioned 7.1 Å away from the guanidinium group of R349 (Figure 1d).^17^ However, a study by Drawz et al. reported an unexpected result, where R349A substitution in PDC-3 significantly decreases LP06 binding affinity, leading to a 52-fold increase in its *K*_i_.^18^ This finding seems counterintuitive, but our study offers a clear and compelling explanation. Along Path 1, R349 forms hydrogen-bond or salt-bridge interactions with LP06 during the early stages of binding (Figure 4a,d). In line with this, energy decomposition analysis shows that P345, N346, and R349 make substantial contributions to the binding free energy, with R349 being especially important (Figures S8 and S9). Within the precovalent ensemble, representative structures from the final stages of three successful trajectories (Replica 4, Replica 6, and Replica 10) exhibit close overall agreement with the crystallographic pose, although LP06 remains marginally less deeply inserted than in the covalent structure (Figure 3h). In this configuration, the LP06 carboxylate is positioned to form a salt-bridge with R349 (Figure 3g), and the resulting precovalent complex (Cluster J) displays a notably favorable binding free energy of −9 kcal/mol. In contrast, the crystallographic structure represents the postreaction covalent adduct formed following nucleophilic attack of S64 on LP06. The covalent bond formation is accompanied by a reorganization of the ligand, displacing the carboxylate from the R349 guanidinium group (Figure 1d). Consequently, the principal functional role of R349 arises along the kinetic binding pathway rather than in stabilization of the final covalent bound state. This mechanistic insight explains why the R349A markedly reduces LP06 binding affinity, despite the substantial spatial separation between the R349 guanidinium and LP06 carboxylate observed in the covalent crystal structure.

Moreover, the hydrophobic interactions with residues in the P2 loop (L119 and Q120), the Ω loop (V211 and Y221), and the R2 loop (A292 and L293) play essential roles in the ligand recognition during the binding process. Prior work has mostly examined how substitutions result in significant changes in the activity of β-lactamases.^38–43^ Existing studies have focused primarily on how alterations at these sites lead to conformational changes in the protein that subsequently affect its activity. However, there seems to be a lack of research investigating how these specific amino acid residues directly influence the binding process of β-lactamase inhibitors. Our study fills the gap by systematically characterizing how these hydrophobic regions contribute to ligand recognition at the molecular level. Therefore, the introduction of additional hydrophobic groups into the ligand structure could enhance these interactions, thereby improving the initial recognition efficiency and binding stability. To assess the generality of these hydrophobic nodes, we performed a multiple-sequence alignment of 6688 unique class C β-lactamase sequences obtained from the BLDB.^5^ Remarkably, all six hydrophobic hotspot positions that we identify show >90% sequence identity across the data set (Figure S10 and Supporting Information 1). This high conservation suggests that the insights obtained from our study on PDC-3 should apply to almost all class C β-lactamases, providing a solid basis for designing broad-spectrum inhibitors that target these shared hydrophobic recognition regions.

### Kinetic Traps Around the Binding Site

Together with the successful binding events observed in some trajectories, several kinetic traps were identified. These traps correspond to meta-binding states in which the ligand stabilizes but does not progress to the optimal bound conformation. Analysis of trajectory data revealed that certain replicas became trapped in conformations within clusters A, C, D, K, L, and M (Figures 5 and S7). These conformations are positioned within the active site, deviating from the crystal-like orientation, or are in the R2 site of PDC-3, which binds the R2 side chain of cephalosporins. Clusters D, K, and M exhibit lower binding free energy values Δ*G*_bind_ compared to the crystalline conformation in cluster J. Specifically, the average binding free energies for Clusters D, K, and M are −31 kcal/mol, −32 kcal/mol, and −27 kcal/mol, respectively, which are lower than that of Cluster J (−23 kcal/mol). It is important to note that cluster J represents a productive configuration that adopts a geometry capable of forming a reversible covalent tetrahedral adduct with S64. Its decisive stabilization therefore comes from subsequent covalent bond formation and the associated electronic and structural reorganization. These contributions are outside the scope of standard MM/PBSA. As a result, the Δ*G*_bind_ for cluster J can appear less negative than those of strongly hydrogen-bonded meta-binding traps such as clusters D, K, and M. Therefore, MM/PBSA can underestimate the stability of the productive state when a chemical step is involved. MM/PBSA was used here to compare the relative noncovalent stabilization across clusters and to identify the interaction patterns and residue contributions that stabilize these states, thereby providing actionable guidance for inhibitor design.

**Figure 5:**
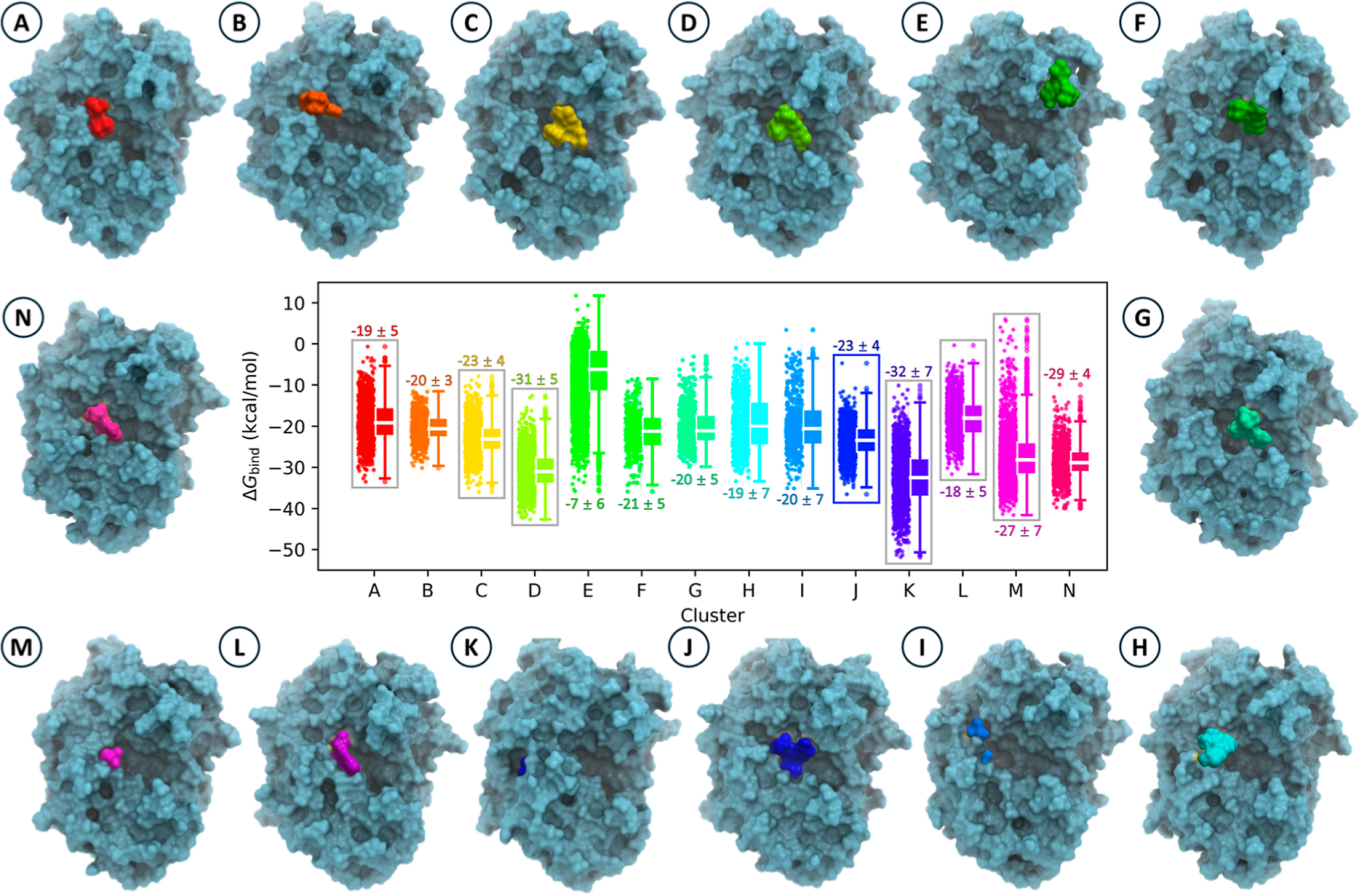
Localization of LP06 across different clusters in PDC-3 and their corresponding binding free energy (Δ*G*_bind_) distributions. (A–N) Representative structures of LP06 bound PDC-3 in each cluster. The violin–box plots combine violin plots (depicting the distribution and density of Δ*G*_bind_) with box plots (showing medians and interquartile ranges). The numbers above or below each violin-box plot represent the mean ± standard deviation Δ*G*_bind_ for each cluster. Clusters highlighted with gray boxes are designated as the final states of LP06 observed across all 10 trajectories.

Insights into these kinetic traps can be gained by examining the representative structures from Clusters D, K, and M. In Cluster D, the ligand occupies the same position as in the crystal structure; however, the orientation is inverted. Specifically, the boronic acid group and carboxyl group swap positions (Figure 4g). Residues such as S64, K67, Y150, and N152 provide significant contributions to the binding free energy Δ*G*_bind_ in conformations of Cluster D, as indicated by their notably low energy values (Figure S9). However, these interactions stabilize the ligand in an orientation incompatible with forming a covalent bond at the active site. In Cluster K, the ligand becomes trapped between the R2 loop and Helix 10, adopting an exceptionally stable conformation. The carboxyl group of LP06 forms hydrogen bonds with Y274, while the boronic acid group engages in hydrogen bonding with R148. The amino group forms hydrogen bonds with N287, and M291 establishes hydrophobic interactions with the dimethyl group of LP06, further stabilizing the ligand within this confined space (Figure 4h). Y274, R148, and M291 emerge as significant contributors to Δ*G*_bind_, anchoring LP06 in this stable but unproductive conformation (Figure S9). In cluster M, the amino group of LP06 forms hydrogen-bonding interactions with N346 and Q284, while the boronic acid group establishes hydrogen bonds with R148 and E272. The carboxyl group interacts electrostatically with the side chain of R349 (Figure 4i). The residues N346, R349, and R148 make substantial contributions to Δ*G*_bind_ (Figure S9), further stabilizing the ligand in this kinetic trap. In addition to clusters D, K, and M, clusters A, C, and L also correspond to unproductive meta-binding states in which LP06 is stabilized by local polar contacts but does not progress toward the precovalent state. Although their Δ*G*_bind_ values are less negative than those of clusters D, K, and M, these states are mechanistically consistent with the same hydrogen-bonding locking principle (Figure S11).

In the crystal structure, LP06 forms a covalent bond with the catalytic residue S64 and establishes hydrogen bonds with S64, Q120, Y150, N152, and S318, and its dimethyl groups participate in hydrophobic interactions with L119, A292, and L293 (Figure 1d).^17^ These observations indicate that strengthening the hydrogen bonding network around the covalent bond formation site can improve the ligand stability and promote covalent bond formation. However, the conformations from clusters A-M exhibit hydrogen bonding interactions that stabilize the ligand but differ significantly from those observed in the crystal structures. We refer to these interactions as hydrogen-bonding locks, defined here as hydrogen bonds that stabilize the ligand in unproductive conformations and prevent its progression to the precovalent binding state. Although hydrogen bonds generally contribute to ligand stability, they can sometimes be counterproductive by locking the ligand in meta-binding sites.^44,45^ This highlights the dual role of hydrogen bonds.

In addition, kinetic traps are typically caused by the difficulty in overcoming kinetic barriers, which can hinder the ligand from entering the active site, leading to bottleneck effects and limiting its activity. For instance, in the study by Dror et al., during the process of drug binding to the β_1_-adrenergic receptor (β_1_AR) and β_2_-adrenergic receptor (β_2_AR), the ligand must cross the barrier between the vestibule and the binding pocket. Failing to do so results in the ligand remaining trapped in metastable states.^46^ Such processes often require protein flexibility. In this case, structural adjustments, such as the separation of Y308 and F193, are necessary to allow the ligand to pass through. Similarly, in the conformations represented by clusters A, C, D, K, L, and M in our study, for the ligand to access the catalytic site, it must either abandon its current position and find an alternative pathway or rely on significant structural adjustments. This demonstrates why active site flexibility is a critical factor in the enzymatic catalytic efficiency. The dynamic characteristics of active sites play a key role in designing more effective enzyme-targeted drugs.^47^

### Analysis of Escape Pathways

To investigate the unbinding of LP06 from PDC-3, three independent simulations using the SMD were performed. In all three trajectories, the ligand dissociates from the binding site within 10 ns, with the COM distance between the ligand and the binding site exceeding 30 Å (Figure 6a and Supporting Information 3). Interestingly, based on the binding free energies, the initial conformations closest to the crystal structure do not exhibit the lowest binding free energies. This observation is consistent with the findings of the binding simulations, where cluster J, the cluster with conformations most similar to the crystal structure, did not have the lowest binding free energies (Figures 5 and 6b). Furthermore, analysis of residue contacts within 4.5 Å over time and their frequency revealed that, in addition to catalytic site residues, the most frequently involved residues are in Helix 10 and Helix 11. These include T289, L293, A292, S288, P290, and N287 on Helix 10, as well as N346, N343, R349, and P345 on Helix 11 (Figures S12 and S13a,b). Furthermore, residues in the Ω loop (V211 and Y221) and the P2 loop (L119 and Q120) also showed high contact frequencies. However, from the contact map and the decomposition of the binding free energy contributions (Figure S13c), it is evident that while LP06 interacts strongly with residues in the Ω loop and P2 loop during the early stages of the unbinding process, these interactions are rapidly weakened as the ligand begins to dissociate from the catalytic site. This suggests that these residues play a crucial role in stabilizing the ligand within the catalytic site.

**Figure 6:**
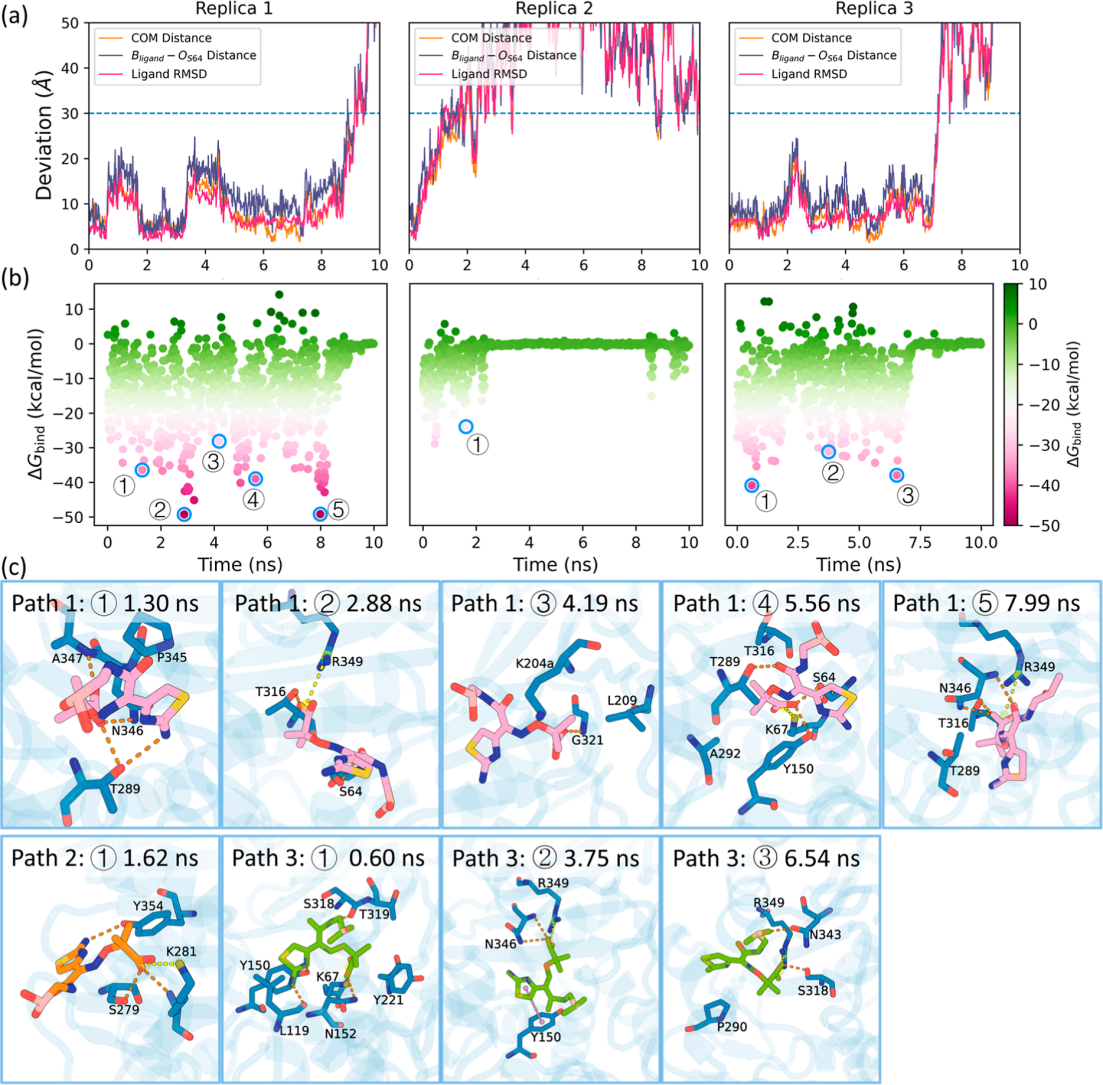
Analysis of LP06 unbinding pathways from PDC-3 across three independent replicas. (a) Time evolution of key geometric parameters across 3 replicas of LP06 unbinding from PDC-3, including the COM distance between the ligand and binding site identified by NanoShaper, B_ligand_–O_S64_ distance, and ligand RMSD (aligned to protein Cα atoms). (b) Binding free energy Δ*G*_bind_ calculated over time. Key points in the simulation corresponding to significant structural transitions or interactions are highlighted and labeled. (c) Structural representations of the ligand at selected time points during the unbinding process. The time windows in which the complex undergoes pronounced conformational changes were identified based on the time evolution of key geometric descriptors in panel (a). Within each transition window, the frame with the lowest binding free energy was selected as the representative structure. Pink ligands correspond to Replica 1, the orange ligand to Replica 2, and the green ligand to Replica 3. Key interactions are indicated: hydrogen bonds are shown in orange, salt bridges in yellow, and π–π stacking in violet. The important residues involved in the unbinding process are shown in stick representation.

Upon analysis of the unbinding trajectories, similar patterns emerge in the unbinding pathway of LP06 from PDC-3. At the beginning of the simulations, the heat maps of energy decomposition (Figure S13c) illustrate that the ligand forms stable and energetically favorable interactions with several key residues within the catalytic motifs in all three replicas. These residues, including G63, S64, K67, Y150, N152, and G317 exhibit consistently negative interaction energies. Additionally, S318, Y221, N320, and R349 also contribute significantly to the binding free energy. From these three replicas, it can be observed that S318 frequently forms hydrogen bonds with the boronic acid group of LP06, while Y221 engages in hydrophobic interactions with the ligand’s thiazole group. R349 establishes a stable salt bridge with the ligand, exhibiting consistently low energy values across multiple frames in all three replicas. Furthermore, N320 occasionally forms hydrogen bonds with the amino group of LP06. As the simulation progresses, the interactions between the ligand and residues in the conserved catalytic motifs, Ω loop, and P2 loop weaken, reflected by increasing energy values, signaling the gradual unbinding of the ligand from the catalytic site. Simultaneously, interactions with residues on Helix 10 and Helix 11 strengthen. For instance, residues in the R2 loop, such as S288, T289, P290, A292, and L293, alongside Helix 11 residues including N343, P345, N346, A347, and R349, play pivotal roles in facilitating the transition of the ligand from the binding site to the protein surface. As the interactions between the ligand and these residues weaken further, the ligand begins to drift away from the protein, and the binding free energy increases sharply until the ligand eventually escapes from the protein.

Although triplicate simulations show similar modes in their unbinding pathways, they are not identical (Figure 6c). In Replica 2, after the ligand leaves the binding site, its carboxyl group forms a transient salt bridge with K281 on the protein surface. This interaction is short-lived, and the ligand quickly dissociates from the protein. In Replica 3, at the beginning of the unbinding process, the thiazole group of LP06 tilts toward the R2 site of PDC-3. The nitrogen atom of thiazole forms a hydrogen bond with the hydroxyl group of Y150, while the amino group forms a hydrogen bond with the backbone of L119. Simultaneously, the carboxyl group establishes a strong salt bridge with K67. These interactions contribute to a significantly low binding free energy between LP06 and PDC-3. Shortly afterward, the ligand temporarily distanced itself from the binding site. However, its carboxyl group is then attracted by the side chain of R349, drawing the ligand back to the R2 loop. Furthermore, the carboxyl group of LP06 forms hydrogen bonds with N346, while the thiazole group engages in π-π-stacking interactions with Y150. The ligand remains stabilized near the R2 loop for a short period before ultimately dissociating completely from the protein.

The simulation of Replica 1 reveals an intriguing trajectory. Initially, LP06 is gradually drawn away from the catalytic site by residues on Helix 10 and Helix 11. However, around 2 ns into the simulation, it unexpectedly reverses direction and re-establishes a salt bridge with R349, temporarily pulling the ligand back into the binding site. Following this, LP06 once again begins to move toward Helixes 10 and 11, drifting further away from the catalytic site. Subsequently, the ligand is briefly attracted by K204a but then subsequently moves away from the binding site. Although it temporarily exits, the ligand is quickly pulled back into the binding site due to electrostatic attraction between its carboxyl group and the positively charged side chain of K67 in the catalytic site. Notably, the orientation of LP06 at this stage differs significantly from that of the crystal structure. Eventually, LP06 is again drawn toward the residues on Helix 10 and Helix 11. During this phase, the carboxyl group forms a highly stable salt bridge with R349, which plays a key role in stabilizing the ligand. Once this salt bridge breaks, the binding free energy Δ*G*_bind_ between LP06 and PDC-3 increases sharply, leading to rapid unbinding of the ligand from the protein. This consistent pattern emphasizes the important contribution of R349 to preserving LP06 engagement within the binding site. Previous work has similarly identified R349 as a key residue in the stabilization of β-lactams and BLIs within the catalytic site.^48^ We posit that R349 acts as a retention site, temporarily stabilizing LP06 prior to its full dissociation (Supporting Information Movie 2). The presence of this kinetic barrier suggests that R349 variants may significantly alter the binding kinetics of BLIs, which is consistent with previous studies showing that R349A substitutions dramatically reduce inhibitor affinity.^18^

### R349A Substitution Alters Substrate Kinetics

To assess the impact of R349A and support the computational analysis above, we measured steady-state kinetics with nitrocefin (NCF) across pH 5.2–9.5 (Table 1). We observed parallel increases in *V*_max_ and *k*_cat_ for both wild-type PDC-3 and the R349A variant. For PDC-3, *k*_cat_ rises from 77 ± 7 s^–1^ at pH 5.2 to 267 ± 26 s^–1^ at pH 9.5 (∼3.5 fold); the corresponding values for R349A are 21 ± 1 to 122 ± 4 s^–1^ (approximately 5.8-fold). These pH trends are consistent with the protonation behavior of the general base K67. At low pH, K67 is predominantly protonated and cannot efficiently abstract the proton from the catalytic S64; as pH increases toward and above K67 p*K*_a_, the fraction of deprotonated K67 grows, accelerating acylation/deacylation until a plateau is reached. Notably, at any pH, the *V*_max_ and *k*_cat_ of the wild-type remain significantly superior to those of R349A (*k*_cat_ of R349A/*k*_cat_ of wild-type = 0.27–0.56), indicating that the positive charge of R349 plays a critical role in stabilizing the acyl-enzyme intermediate or transition state. The results observed here, taken together with the simulations, suggest that R349 promotes efficient chemical conversion and provides the necessary retention for the substrates. The R349A substitution alters the structural interactions, reducing acyl-enzyme intermediate or transition state stabilization and possibly allowing premature substrate dissociation, thereby suppressing the overall turnover rate.

**Table 1:** Steady–State Kinetic Parameters for Nitrocefin Hydrolysis by Wild-type PDC-3 and R349A at Different pH Values

enzyme	parameter	pH 5.2	pH 7.2	pH 8.0	pH 9.5
wild-type PDC-3	*V*_max_(μM s^–1^)	0.69 ± 0.07	1.37 ± 0.10	2.00 ± 0.20	2.40 ± 0.20
	*k*_cat_(s^–1^)	77 ± 7	152 ± 15	222 ± 20	267 ± 26
	*K*_m_(μM)	25 ± 1	28 ± 1	24 ± 1	21 ± 1
	*k*_cat_/*K*_m_(μM^–1^s^–1^)	3.1 ± 0.3	5.4 ± 0.5	9.3 ± 0.9	12.7 ± 1.2
R349A	*V*_max_(μM s^–1^)	0.23 ± 0.01	0.93 ± 0.03	1.25 ± 0.02	1.34 ± 0.04
	*k*_cat_(s^–1^)	21 ± 1	85 ± 3	114 ± 2	122 ± 2
	*K*_m_(μM)	4.0 ± 0.7	10.1 ± 1.5	12.6 ± 1.1	10.8 ± 1.6
	*k*_cat_/*K*_m_(μM^–1^s^–1^)	5.3 ± 0.9	8.4 ± 1.3	9.0 ± 0.8	11.3 ± 1.7

In contrast, *K*_m_ is uniformly lower for R349A (e.g., pH 5.2: 4.0 ± 0.7 vs 25 ± 1 μM; pH 9.5: 10.8 ± 1.6 vs 21 ± 1 μM), yielding apparent efficiencies *k*_cat_/*K*_m_ that exceed wild-type at acidic pH ( pH5.2:5.3±0.9 vs 3.1 ± 0.3 μM^–1^s^–1^;pH7.2:8.4 ± 1.3 vs 5.4 ± 0.5) and are slightly lower at basic pH(pH8.0:9.0±0.8 vs 9.3±0.9;pH9.5:11.3±1.7 vs 12.7 ± 1.2). The lower *K*_m_ observed for R349A is most readily explained by the substantial reduction in *k*_cat_ in the Briggs–Haldane relationship *K*_m_=(*k*_‑1_+*k*_cat_)/*k*_1_. It is also noteworthy that the *K*_m_ of wild-type PDC-3 shows a slight increase near pH≈7 and then decreases as pH continues to rise. This behavior likely reflects the progressive deprotonation of K67, which weakens the electrostatic attraction at the binding site. The persistent positive charge on R349 compensated for the loss. In R349A, which lacks this compensatory mechanism, the binding site’s attraction to NCF is weakened, reducing substrate entry efficiency and again highlighting R349’s role as a crucial anchor in guiding the substrate and stabilizing the complex.

It is notable that the arginine anchor residue (R349) identified in this study is highly conserved and present in over 99% of 6888 unique class C β-lactamase sequences (Figure S11). This strong evolutionary conservation suggests that the functional role we observed for R349 is broadly representative of the class C β-lactamase family. Beyond class C enzymes, structural alignments of representative serine β-lactamases from different classes (Figure S14; class A KPC-2, PDB ID: 5MGI; class C PDC-3, PDB ID: 8SDR; and class D OXA-24/40, PDB ID: 5TG5) further support the generalizability of this mechanistic model. In addition to the expected similarities in their conserved catalytic motifs, structural superimposition reveals that the position corresponding to R349 in PDC-3 is occupied by arginine residues in other classes as well (R220 in KPC-2; R351 in OXA-24/40, numbering based on SAND^49^). Together, these sequence- and structure-level observations support a broadly applicable mechanistic framework in which a conserved arginine residue serves as a key anchor.

## Conclusions

MDBind simulations identified three distinct pathways of LP06 binding to PDC-3, each characterized by unique intermediate states and interactions. Residues within the P2 loop (L119 and Q120), the Ω loop (V211 and Y221), and the R2 loop (A292 and L293) play pivotal roles in guiding the ligand to its catalytically active state. These residues engage primarily in hydrophobic interactions with LP06, which are crucial for the initial recognition of ligands and early stage stabilization. Furthermore, several kinetic traps were identified. In these traps, the ligand is stabilized in orientations that are incompatible with catalysis. Hydrogen bonding interactions between LP06 and some residues in PDC-3 effectively lock the ligand in nonproductive states. Although these interactions contribute to the overall stability of the ligand, they also introduce energy barriers that hinder its transition to catalytically active conformations. These findings highlight the dual-edged nature of hydrogen bonding interactions in ligand–protein binding. R349 emerges as a critical residue, serving as an anchor to secure LP06 within the catalytic site and facilitating its optimal position for catalysis. These findings provide valuable insights for rational drug design, particularly in the development of next-generation inhibitors targeting class C β-lactamases. First, enhancing the hydrophobic interactions between inhibitors and key residues in the P2, Ω, and R2 loops could significantly improve the binding efficiency and stability of the inhibitors. Second, it is crucial to avoid introducing polar groups near regions other than the boronic acid group to minimize the risk of nonproductive hydrogen bonding interactions with protein surface residues, which could lead to kinetic traps. Finally, designing modifications to strengthen interactions with R349 could further enhance the anchoring of the ligand and ensure effective engagement within the catalytic site.

In conclusion, this work offers a comprehensive understanding of the dynamic binding and unbinding processes of LP06 to PDC-3, elucidating the molecular mechanisms underlying ligand recognition and stabilization. Remarkably, sequence alignment confirmed that this mode of recognition is conserved across class C β-lactamases, suggesting broad applicability. Hence, this fundamental principle appears applicable across clinically relevant class C β-lactamases, offering a structural basis for the design of broad-spectrum inhibitors.

## Methods

### MD Setup

The crystal structure of PDC-3 bound to LP06 (PDB ID: 8SDR) was obtained from the Protein Data Bank.^17,50^ The bound state of the PDC-3–LP06 complex was directly derived from the crystal structure, with the covalent bond between the oxygen atom of S64 and the boron atom of LP06 disrupted to reflect the noncovalent binding state. For the unbound state, the ligand was positioned at a distance of 30 Å away from the binding site (residues S64, L119, Q120, R148, Y150, Y221, E272, G286, N287, M291, A292, K315, T316, G317, S318, T319, and N346), which were identified by NanoShaper.^51^ The protonation states of residues were assigned using the BiKi Life Sciences suite 1.5 of BiKi Technologies s.r.l.^28^ LP06 was optimized using the Gaussian 16 software package at the Hartree–Fock (HF) level of theory with the 6–31G* basis set. Following optimization, restrained electrostatic potential (RESP) partial charges were computed for the ligand using Gaussian 16.^52^ Ligand parameters were subsequently generated utilizing the Antechamber and Parmchk modules from AmberTools22, applying the general Amber force field.^53^ Specific parameters related to boron atoms were derived from the study conducted by Kurt et al.^54^ The preprocessed protein–ligand complexes were solvated in a periodic cubic box of water molecules represented by the transferable intermolecular potential with 3 points.^55^ Periodic boundary conditions in all directions were utilized to reduce the effects of the finite system.^56^ The boundaries of the water box were set to 15 Å from any protein atom. To neutralize the total charge, Na^+^ or Cl^–^ counterions were added.

The system was simulated using Gromacs 4.6.1 and Plumed 2.0 (BiKi customized versions) and underwent energy minimization with 5000 steps using the steepest descent method.^57,58^ Equilibration comprised four steps: (1) a 0.1 ns simulation under the *NVT* ensemble at 100 K, (2) a 0.1 ns simulation under the *NVT* ensemble at 200 K, (3) a 0.1 ns simulation under the *NVT* ensemble at 300 K, and (4) a 1 ns simulation under the *NPT* ensemble at 300 K. Throughout all steps, the protein backbone and ligand heavy atoms were restrained with a force constant of 1000 kJ/mol/nm^2^. All simulations employed the leapfrog integrator. The Verlet cutoff scheme was used to handle short-range interactions, with a frequency of updating the neighbor list every 20 steps. Electrostatic interactions were treated using the particle mesh Ewald method with a grid spacing of 0.16 nm and a cubic interpolation order of 4.^59^ The Coulomb cutoff distance was set to 1.1 nm, while Lennard-Jones interactions were calculated using a cutoff distance of 1.1 nm.

### MDBind Simulations

The MDBind method within the BiKi suite introduces an additive external force to the regular potential energy of the system in MD simulations.^28^ The bias consists of a moving umbrella, where the force constant *K*(*t*) is adaptively modified over time. The bias is described by $\frac{1}{2}CK\left(t\right){\left(CV\left(t\right)-{CV}_{0}\left(t\right)\right)}^{2}$ . The collective variable CV­(*t*) is the Yukawa potential: $\sum_{l\in L,s\in S}\frac{Q_{l}Q_{s}}{r_{ls}\left(t\right)}\exp\left(-\frac{r_{ls}\left(t\right)}{\lambda }\right)$ , where *r*_ls_(*t*) is the interatomic distance, exp­(−*r*/*λ*), serves as a decay function to avoid unnecessary long-range forces and λ (set to 10 Å) controls the spatial range of the decay. *S* is a set of heavy atoms in the binding site identified by NanoShaper, and *L* is the set of heavy atoms of LP06. The CV_0_(*t*) value evolves over time as in steered MD, and its final value is set heuristically by BiKi. Fictitious charges are placed on the sets of attracting atoms with *Q*_l_ = -*Q*_s_∀*l*∈*L*,*s*∈*S*. The overall intensity is ruled by the *C* parameter. To ensure stability, the modulus of the biasing force is rescaled every 0.2 ps of the simulation to ensure that it does not exceed a predefined fraction (10%) of the actual resultant force acting on the ligand, which originates from the rest of the system. Additionally, the bias is further smoothly reduced based on the distance between *L* and a subset *S* ^′^⊂*S* (the heavy atoms of residue S64). A scaling factor γ, computed as $\gamma =\frac{1}{1+\exp\left(-ss\left(dist‐th\right)\right)}$ , with ss and th representing the steepness and distance threshold of the switching, respectively, multiplies the biasing force (this is a component of *K*(*t*)). The *dist* value is defined as $dist=\underset{y\in S^{\prime },x\in L}{\min d\left(x,y\right)}$ , where *d*(*x*,*y*) is the pairwise distance between the atoms *x*∈*L* and *y*∈*S* ^′^. Once *K*(*t*) has begun to decrease due to the approach of the *S* ^′^ set, it can no longer increase even if the ligand bounces back. This confers to the method a very smooth behavior; the rationale is that after the transition state is overcome (ruled by the *t* threshold), the bias is inactivated. For a smooth variation, *K*(*t*) is time-averaged over a 2 ps circular buffer. The equilibrated unbound state was used as the initial state for the MDBind simulations. A total of 27 replicas were simulated under the *NVT* ensemble at 300 K, with each replica running for 50 ns at a time step of 2 fs.

### Scaled Molecular Dynamics

To investigate the process of unbinding of the ligand from the binding site, we used the SMD method, which introduces a scaling factor (λ) to the potential energy function.^29^ This approach reduces energy barriers and facilitates transitions between states. In this study, the scaling factor was set to λ = 0.4 (Figures S15–S17 and Supporting Information Note 2), effectively lowering the energy landscape to accelerate the sampling of the unbinding pathways. The equilibrated bound state of the complex was used as the initial state for the SMD. A harmonic restraint of 50 kJ/mol/nm^2^ was applied to the protein backbone atoms to maintain structural integrity during the simulations. Exceptions were made for residues located in the binding site that were left unrestrained to preserve flexibility in the vicinity of the ligand-binding site. The setup of the SMD simulations was carried out by using the BiKiNetics module of the BiKi suite. Three replicas were launched, each running for 10 ns under the *NVT* ensemble at 300 K with a time step of 1 fs. The ligand was considered unbound when the distance between the protein COM and ligand COM reached 30 Å.

### Self-Organizing Maps Training and Clustering

A SOM is an unsupervised learning method in which neurons are arranged in a grid map to enforce topological relationships.^33^ This approach effectively visualizes multidimensional input data in a low-dimensional representation. To cluster the conformations during the binding path, we utilized the PathDetect-SOM tool for SOM training, generating a 10 × 10 sheet-shaped SOM with a hexagonal lattice shape and without periodicity across the boundaries.^34^ To train the model, 25 residues that most frequently contacted LP06 (within 4.5 Å) were selected in all binding trajectories. The time-dependent pairwise Euclidean distances between the heavy atoms of the ligand and the heavy atoms of the selected residues were used as input features for SOM training. A capping threshold of 7 Å was applied, where distances exceeding this threshold were set to the capping value. The SOM training was conducted for 5000 iterations. After training, each frame of the simulations was assigned to a neuron on the map, where each neuron represents a configurational microstate of a ligand–protein complex. Subsequently, these neurons were grouped into a representative number of clusters using agglomerative hierarchical clustering with Euclidean distance and complete linkage. All SOM training and analyses were performed within the R statistical environment using the *kohonen* and *igraph* packages.^60,61^

### Binding Free Energy Calculations Using MM/PBSA

The binding free energy (Δ*G*_bind_) of LP06 to PDC-3 was calculated using the molecular mechanics/Poisson–Boltzmann surface area (MM/PBSA) method in gmx_MMPBSA.^62,63^ For each system (complex, receptor, and ligand), the total free energy was estimated as *G*_total_ ≈ *E*_MM_ + *G*_solv_, where *E*_MM_ includes van der Waals (*E*_vdW_) and electrostatic (*E*_ele_) contributions. The solvation free energy (*G*_solv_) was divided into polar and nonpolar components; the polar term was calculated using the Poisson–Boltzmann equation, while the nonpolar term was estimated from the solvent-accessible surface area based on the empirical relation Gnonpolar = γ·SASA + β. The binding free energy was then obtained as Δ*G*_bind_ ≈ *G*_total_^complex^ – (*G*_total_^receptor^ + *G*_total_^ligand^). Additionally, a per-residue energy decomposition analysis was performed to identify the key residues contributing to the binding free energy.

### Structural Analysis and Visualization

The distances and RMSD between the ligand and the protein were calculated using the MDAnalysis library.^64^ Key binding interactions, including hydrogen bonds, salt bridges, hydrophobic interactions, and π–π stacking, were identified using the Protein–Ligand Interaction Profiler (PLIP).^65^ Hydrogen bonds were characterized with a donor–acceptor distance cutoff of 3.5 Å and an angle threshold of ≤30°. Salt bridges were defined as interactions between oppositely charged groups within a distance of 4.0 Å. Hydrophobic interactions were detected as contacts between hydrophobic groups within 4.5 Å, while π–π stacking interactions were analyzed based on a centroid-to-centroid distance of ≤6.0 Å and an angular deviation of ≤30°. PyMOL and ICM-Pro (Molsoft L.L.C.) were utilized to visualize protein–ligand interactions, illustrate binding modes, and generate structural images at different stages of the simulations.

### Steady-State Kinetic Assays

The PDC β-lactamases were cloned, expressed, and subjected to site-directed mutagenesis with modifications to previously described protocols.^66^ Briefly, plasmids containing the expression constructs [pET24a­(+) *bla*_PDC‑3_ and pET24a­(+) *bla*_PDC‑3_ R349A] were transformed into BL21-CodonPlus (DE3)-RP cells. Kinetic measurements for purified wild-type PDC-3 and R349A mutant were performed at room temperature using an Agilent 8453 diode array spectrophotometer with 1.0 cm path length quartz cuvettes. Assays were carried out in buffers adjusted to pH5.2,7.2,8.0 , and 9.5. Hydrolysis of nitrocefin (NCF, a readily hydrolyzed chromogenic cephalosporin) was monitored at 482 nm, using a differential extinction coefficient Δ*ε*_482_ = 17,400M^–1^cm^–1^. Initial velocities (*v*) were determined from the linear portion of the reaction progress curves and fitted by nonlinear least-squares to the Henri Michaelis–Menten equation in Origin 7.5

$$v=\frac{V_{\max}\left[S\right]}{K_{m}+\left[S\right]}$$

Turnover numbers (*k*_cat_) were calculated as *k*_cat_ = *V*_max_/[*E*]_T_, where [*E*]_T_ is the total active enzyme concentration determined from active-site titration.

## Supplementary Material









## Data Availability

All simulation data, representative structures, and analysis scripts are available for download at 10.5281/zenodo.18343108.

## Notes

COM distance for raw replica trajectories, comparison of LP06 with a crystal structure, hierarchical clustering of SOM, convergence of SOM, conformational variability within each cluster, SOM-based cluster assignment, SOM grid projections, frequency histograms, average binding free energy contributions, sequence identity profiles, representative structures of kinetic traps, residue contacts with LP06 unbinding, representative structures from Class A, and D β-lactamases, and time evolution of COM and RMSD with different scaling factors) (PDF)
Multiple sequence alignment (CSV)
LP06 binding movie (MP4)
LP06 unbinding movie (MP4)

## Abbreviations Used

ABF: adaptive biasing force
AmpC: ampicillin-resistant cephalosporinase
BLDB: beta-lactamase database
BLIs: β-lactamase inhibitors
COM: center-of-mass
CTX-M: cefotaxime-hydrolyzing enzyme, Munich
FDA: Food and Drug Administration
GAFF: General Amber force field
HF: Hartree–Fock
IC_50_: half maximal inhibitory concentration
*K*i: inhibition constant
*K*_cat_: turnover number
*K*_m_: Michaelis constant
*k* ^–1^: rate of substrate unbinding
*k*_1_: rate of substrate binding
KPC: *Klebsiella pneumoniae* Carbapenemase
LiGaMD: ligand Gaussian accelerated molecular dynamics
MD: molecular dynamics
MDBind: MD-binding
MetaD: metadynamics
MM/PBSA: molecular mechanics/Poisson–Boltzmann surface area
NCF: nitrocefin
NPT: isothermal–isobaric ensemble (number of particles, pressure, temperature)
NVT: canonical ensemble (number of particles, volume, temperature)
OXA: oxacilliniase
PaCS-MD: parallel cascade selection molecular dynamics
PCA: principal components analysis
PBC: periodic boundary conditions
PDB: Protein Data Bank
PDC-3: *pseudomonas*-derived cephalosporinase-3
PLIP: protein–ligand interaction profiler
PME: particle mesh Ewald
RMSD: root-mean-square deviation
SASA: solvent-accessible surface area
SOM: self-organizing map
SMD: scaled molecular dynamics
SuMD: supervised molecular dynamics
tICA: time-lagged independent components analysis
TIP3P: transferable intermolecular potential with 3 points
*V*_max_: maximum reaction velocity.
