# Supplementary material for: Structural and Kinetic Basis for the Rational Design of Next-Generation β‑Lactamase Inhibitors
Source: J Med Chem. 2026 Mar 16;69(6):7262–75. doi: 10.1021/acs.jmedchem.5c03315 (PMC13036776; doi:10.1021/acs.jmedchem.5c03315)
Supplement: Supplementary file 1 [file jm5c03315_si_001.pdf]

Supporting Information for

Structural and Kinetic Basis for the Rational Design of Next-Generation β-Lactamase Inhibitors

Shuang Chen[1], Muchen Yu[1], Manming Xu[1], Sergio Decherchi[2], Andrea M.Hujer[3], Christopher R. Bethel[3], Robert A. Bonomo[3,4,5,6,7], Shozeb Haider[1,8, *]

[1] Department of Pharmaceutical and Biological Chemistry, School of Pharmacy, University College London, London WC1N 1AX, United Kingdom

[2] Data Science and Computation Facility, Fondazione Istituto Italiano di Technologia, Via Morego 30, Genoa 16163, Italy

[3] Department of Molecular Biology and Microbiology, Case Western Reserve University School of Medicine, Cleveland, OH 44106-5029, USA

[4] Department of Medicine, Case Western Reserve University School of Medicine, Cleveland, OH 44106-5029, USA

[5] Clinician Scientist Investigator, Louis Stokes Cleveland Department of Veterans Affairs Medical Center, Cleveland, OH 44106-1702, USA

[6] Departments of Pharmacology, Biochemistry, and Proteomics and Bioinformatics, Case Western Reserve University School of Medicine, Cleveland, OH 44106-5029, USA

[7] CWRU-Cleveland VAMC Center for Antimicrobial Resistance and Epidemiology (Case VA CARES), Cleveland, OH 44106-5029, USA

[8] Prince Fahd Bin Sultan Chair for Biomedical Research (PFSCBR), University of Tabuk, Tabuk 71491, Saudi Arabia

*Corresponding Author
Prof Shozeb Haider (shozeb.haider@ucl.ac.uk)
ORCID: 0000-0003-2650-2925

Supplementary Note 1

Numerous crystal structures of LP06 in complex with β-lactamases are available in the Protein Data Bank (PDB), spanning multiple clinically relevant enzymes. These include TEM-1 variants (G238A, PDB ID: 1JWV; M182T, PDB ID: 1M40), CTX-M family enzymes (CTX-M-9, PDB ID: 1YLY; CTX-M-14, PDB ID: 1YLZ), PenL variants (C69Y, PDB ID: 6AFN; N136D, PDB ID: 6AFP), SHV-1 (PDB ID: 3MKE) and FOX-4 (PDB ID: 5CHM), as well as PDC-3 (PDB ID: 8SDR) and its Y221H variant (PDB ID: 8SDS).

Supplementary Note 2

We determined $\lambda = 0.4$ based on a set of SMD runs at $\lambda = 0.2$, 0.4, and 0.6 (Figure S15-S17). At $\lambda = 0.2$, unbinding was nearly instantaneous in all replicas (<1 ns) but accompanied by large, nonphysical Cα deviations (RMSD ≈ 3 Å), indicating over-aggressive scaling and a high risk of artifacts. In addition, the system blowed up shortly after the ligand moved away from the protein in all replicas. At $\lambda = 0.6$, protein structure was preserved (Cα RMSD ≈ 1 Å). The unbinding typically required ~20 ns and. At $\lambda = 0.4$, unbinding was consistently achieved within 10 ns across replicas while the protein remained similar to the crystal structure (Cα RMSD ≈ 1 Å), striking a good balance between acceleration and structural fidelity. Given that $\lambda = 0.40$ and $\lambda = 0.60$ showed comparable preservation of protein structure, but $\lambda = 0.40$ allowed unbinding events to be observed more rapidly, we selected $\lambda = 0.40$ for our production of SMD simulations.

Supplementary Data 1 Multiple sequence alignment of class C β-lactamases from BLDB.

Supplementary Data 2 Movie of LP06 binding to the binding site of PDC-3.

Supplementary Data 3 Movie of LP06 unbinding from the binding site of PDC-3.

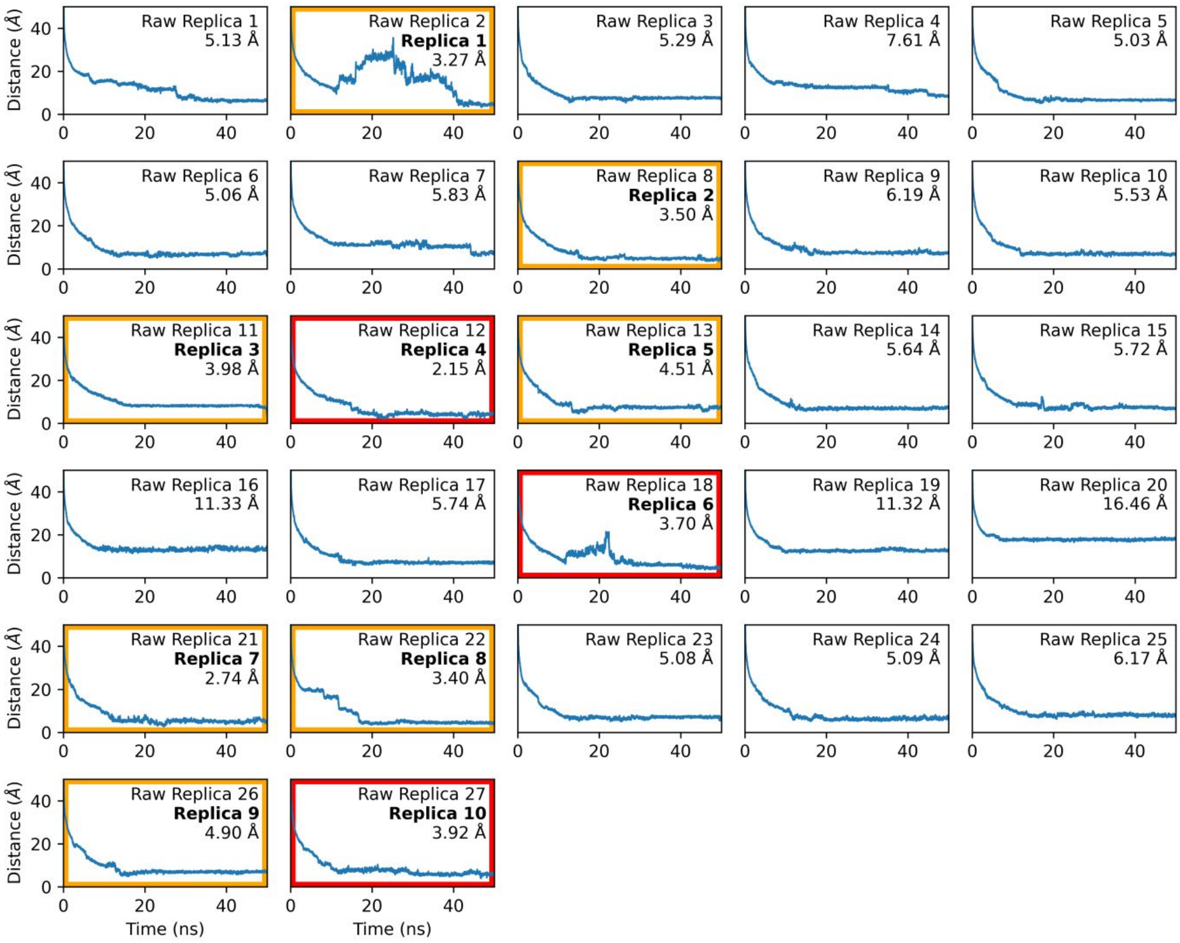

Figure S1. Time evolution of the center-of-mass (COM) distance between the ligand and the binding site is shown for each raw replica (Raw Replica 1–27). Trajectories where the ligands successfully entered the binding sites (COM distance < 5 Å) are highlighted with orange/red borders (Replica 1–10), while replicas achieving conformations closely resembling the crystal structure of PDC-3 bound to LP06 (RMSD values < 2 Å) are highlighted with red borders. The minimum COM distance for each replica is annotated in Å.

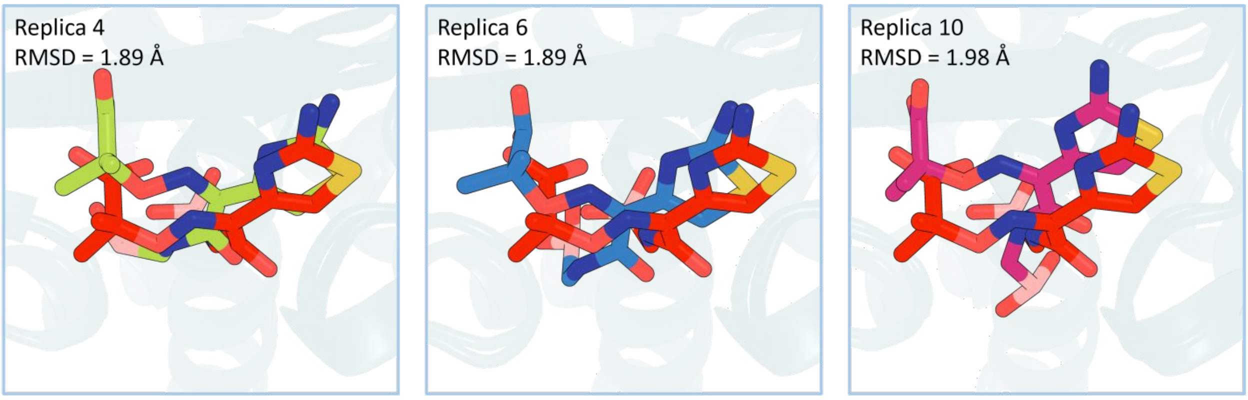

Figure S2. Superimposition of LP06 conformations from simulations with the crystal structure. The red ligand represents the crystal structure, while yellow, purple, and blue correspond to the minimum ligand RMSD conformations from Replica 4, Replica 6, and Replica 10, respectively. All proteins are shown in a transparent cartoon representation. The ligand RMSD values, calculated by aligning protein Cα atoms to the crystal structure, are 1.89 Å (Replica 4 and 6) and 1.98 Å (Replica 10), using the crystal structure as the reference, demonstrating close resemblance to the crystal structure.

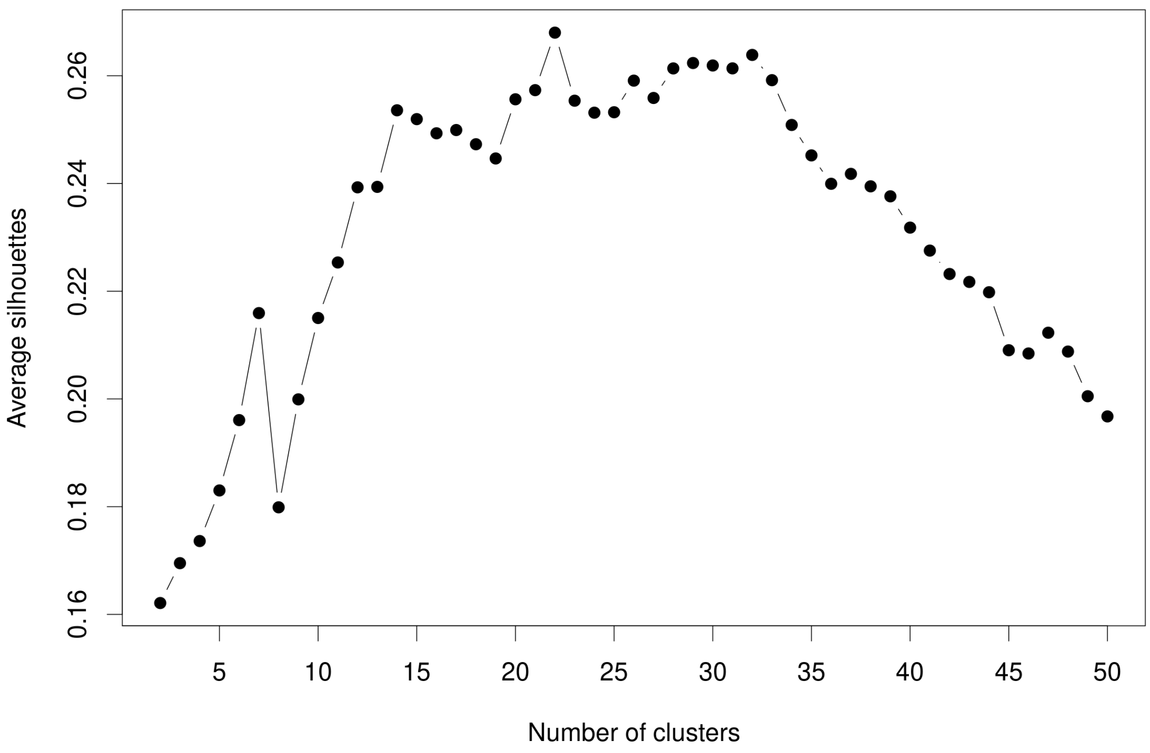

Figure S3. Average silhouette score versus the number of clusters (k = 2–50) for hierarchical clustering of SOM codebook vectors. The selected k=14 marks the onset of the plateau and was used for SOM-based analysis.

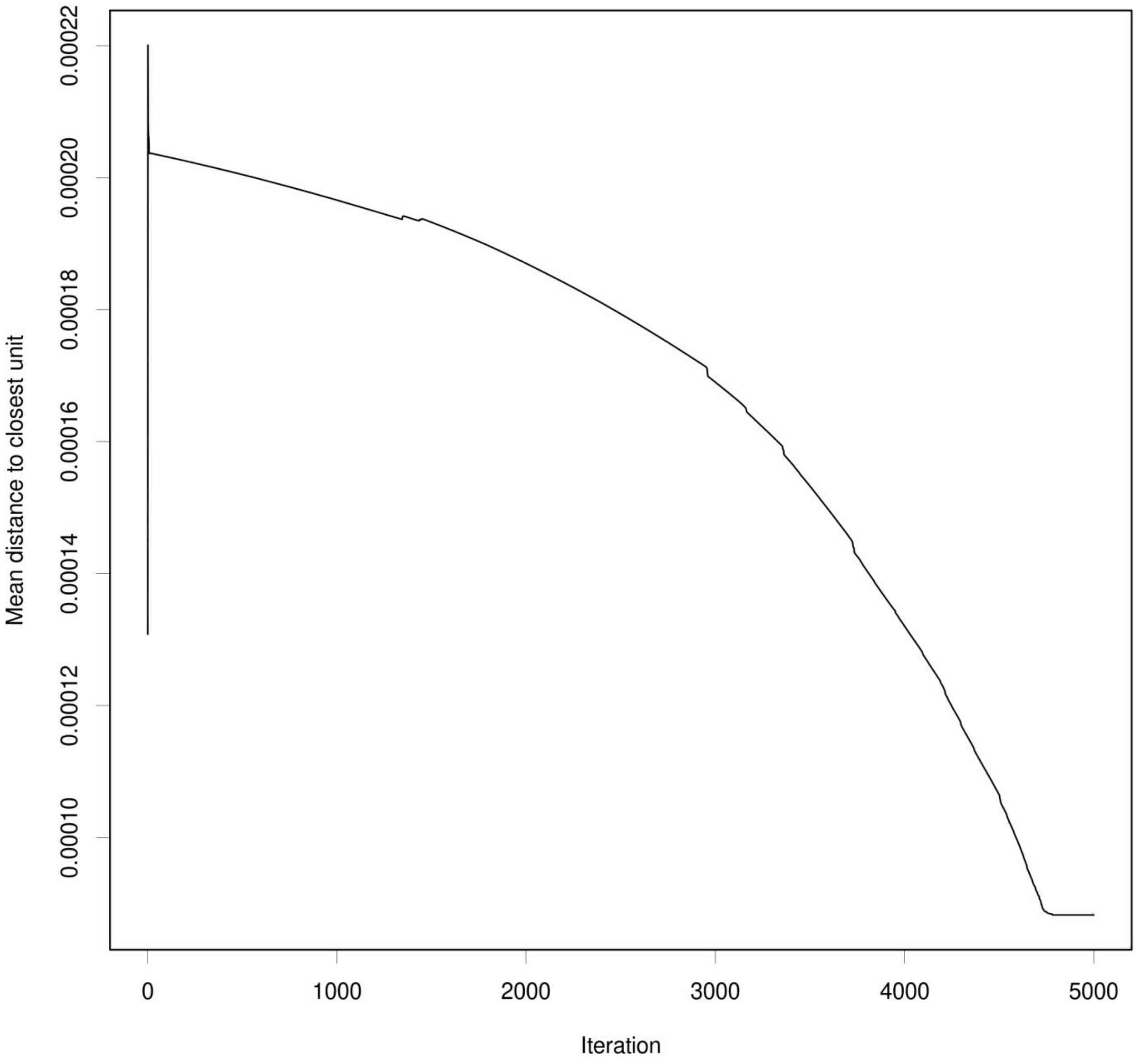

Figure S4. Convergence of the Self-Organizing Map (SOM) training process. The y-axis represents the mean distance to the closest unit, while the x-axis indicates the number of iterations. The decreasing trend demonstrates the progressive refinement of the SOM, with distances stabilizing towards the end, indicating convergence.

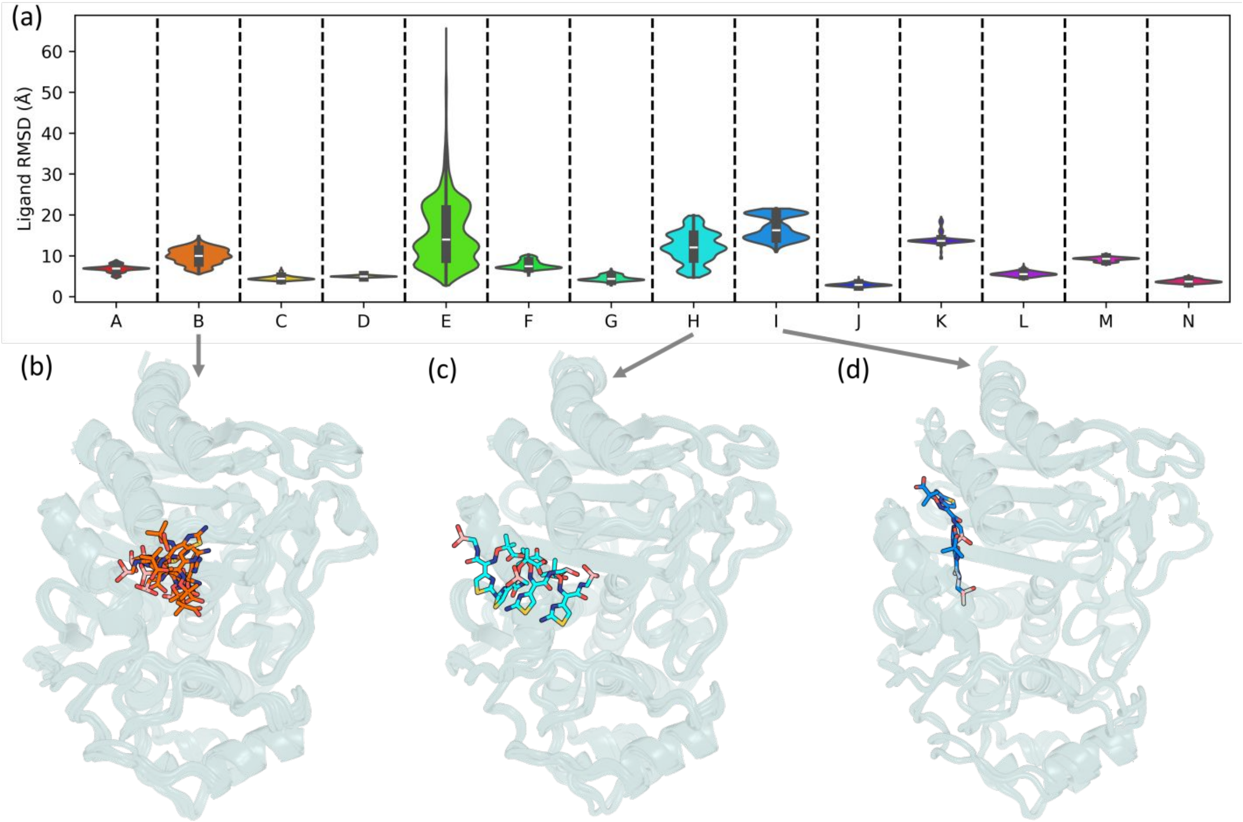

Figure S5. Conformational variability within each cluster. (a) The ligand RMSD distribution across 14 clusters. Protein Cα atoms were used for alignment and the crystal structure (PDB ID: 8SDR) was used as the reference. (b-d) Representative structures of the nodes from Cluster B (b), H (c) and I (d).

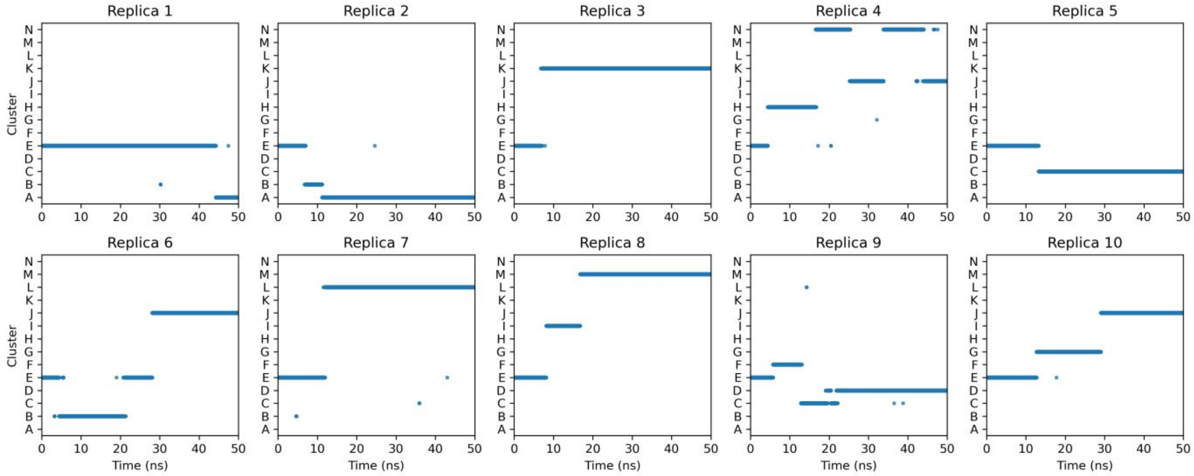

Figure S6. Time evolution of SOM-based cluster assignments across 10 replicas. Each point corresponds to a sampled frame from the respective trajectory and is mapped to its assigned cluster.

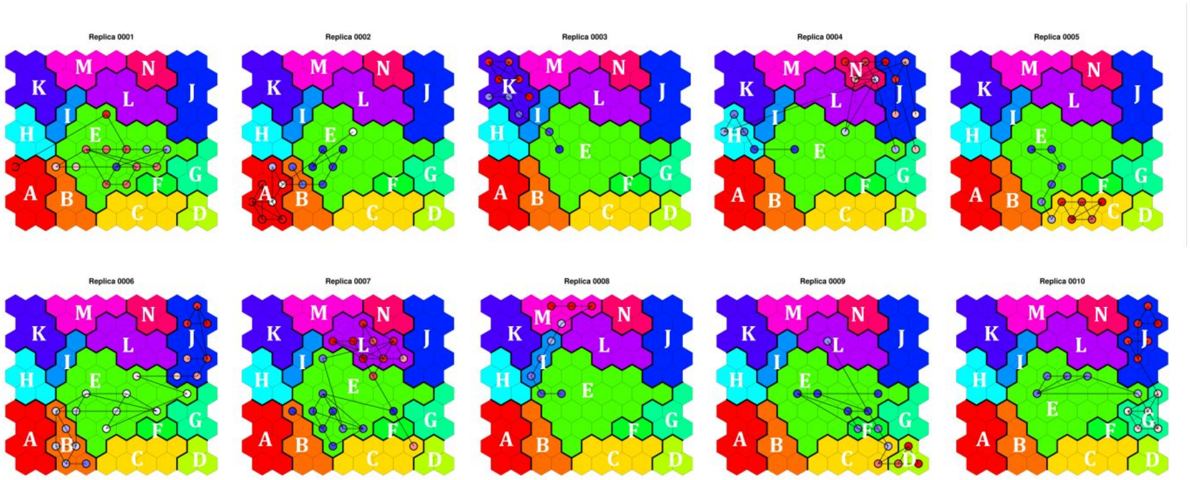

Figure S7. SOM grid projections of the binding pathways for each replica simulation of LP06 binding to PDC-3. Each hexagon represents a SOM node, and the transitions observed during the simulations are traced by black lines connecting the nodes. These pathways illustrate the progression of the ligand from the initial states to the final states.

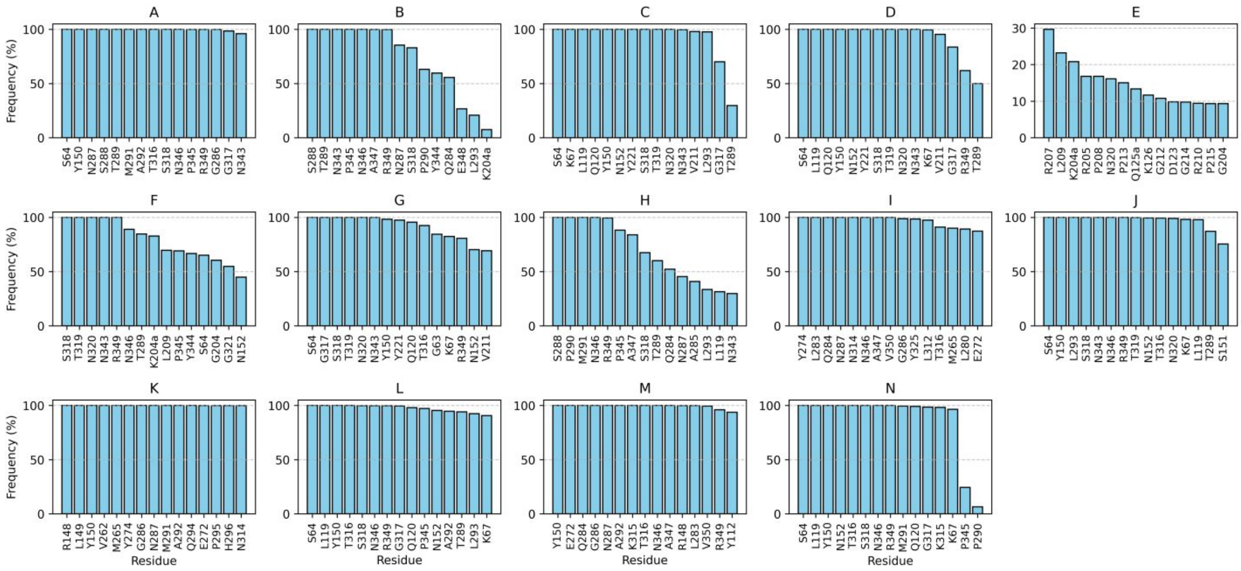

Figure S8. Frequency histogram depicting the top 15 residues most frequently in contact with LP06 (< 4.5 Å) for each representative node (the one with the highest frame population) from SOM clusters (A) 1, (B) 2, (C) 3, (D) 4, (E) 5, (F) 6, (G) 7, (H) 8, (I) 9, (J) 10, (K) 11, (L) 12, (M) 13, (N) 14.

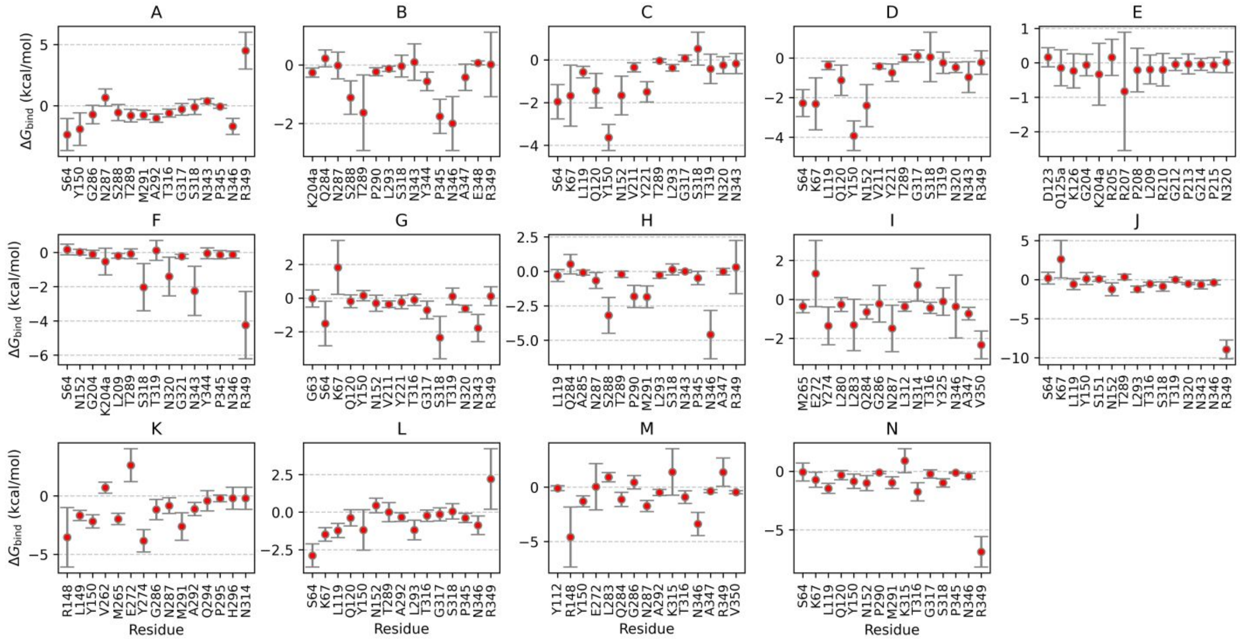

Figure S9. Average binding free energy contributions (ΔG_{bind}) of the top 15 residues with the highest frequency of contacts (within 4.5 Å) within each representative node (the one with the highest frame population) from each SOM clusters (A) 1, (B) 2, (C) 3, (D) 4, (E) 5, (F) 6, (G) 7, (H) 8, (I) 9, (J) 10, (K) 11, (L) 12, (M) 13, (N) 14. Error bars represent the standard deviations. Residues with lower binding free energies ($\Delta G_{bind} < 0$) indicate favorable interactions contributing to ligand stability in the binding site.

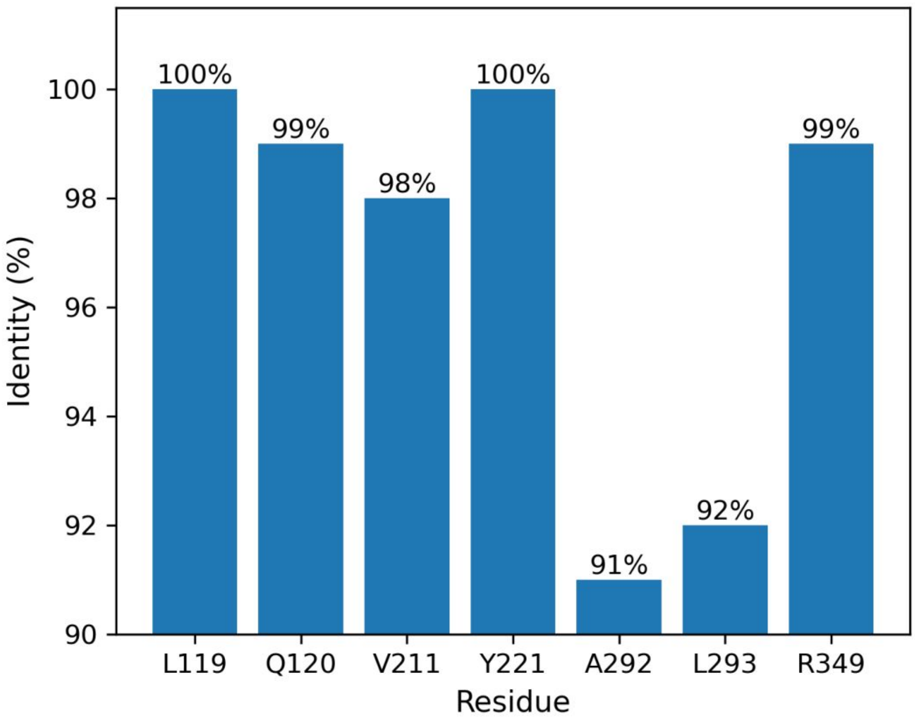

Figure S10. Sequence-identity profile for the hydrophobic-recognition residues identified in PDC-3 across 6,688 class C β-lactamase sequences retrieved from BLDB (31 July 2025).

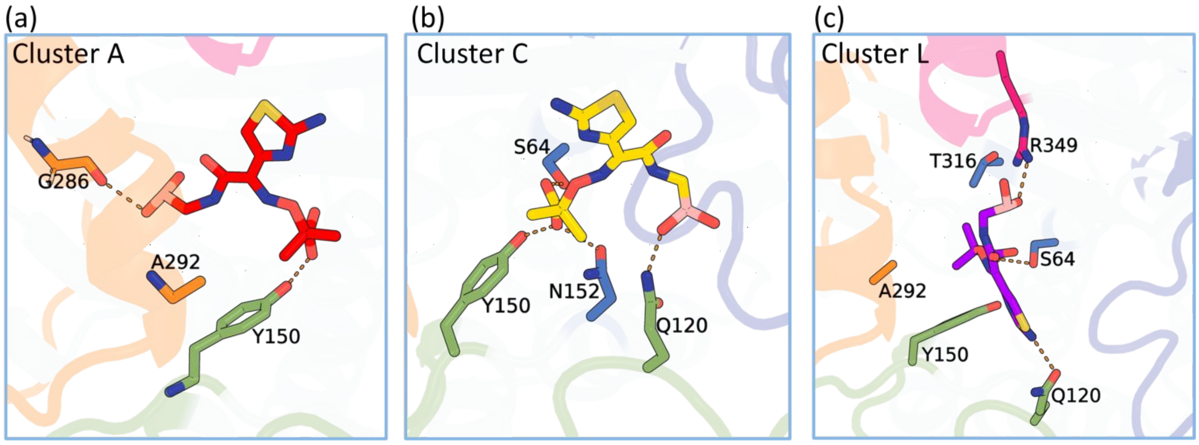

Figure S11. Representative structures of kinetic traps from Cluster A, C and L. The Ω-loop is shown in purple, the R2 loop in orange, the P2 loop in green, and Helix 11 in pink. Salt bridges are shown as orange dashed lines, and hydrogen bonds are shown as yellow dashed lines.

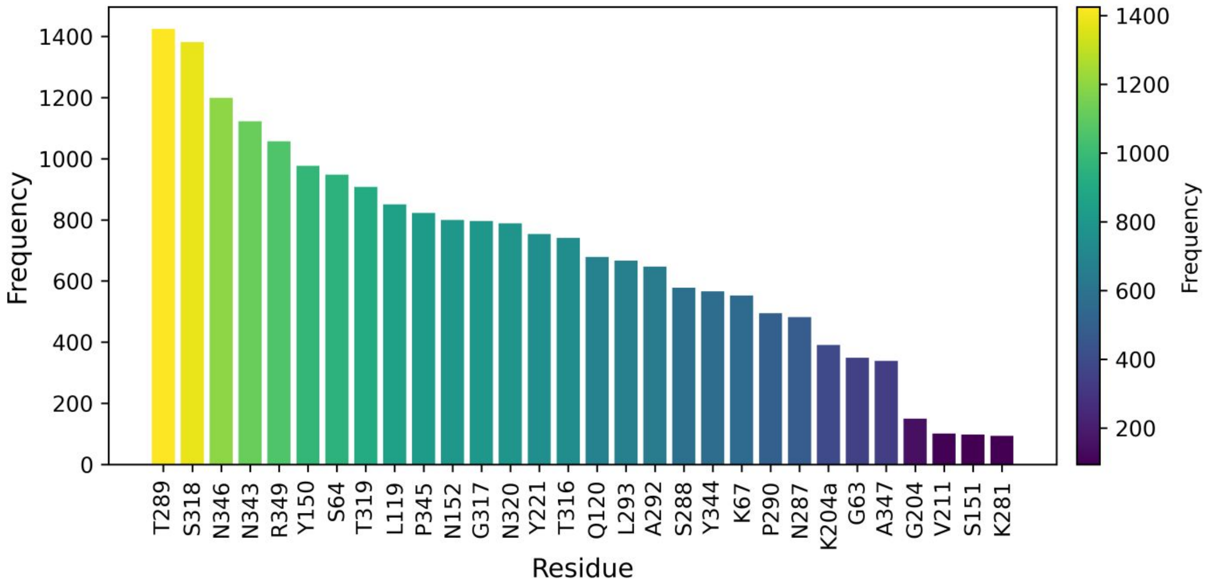

Figure S12. Cumulative frequency of residue contacts with LP06 during unbinding. The histogram represents the total frequency of residues within 4.5 Å of LP06, aggregated across three replicas. The top 30 residues are shown, with the color bar indicating the corresponding contact frequency.

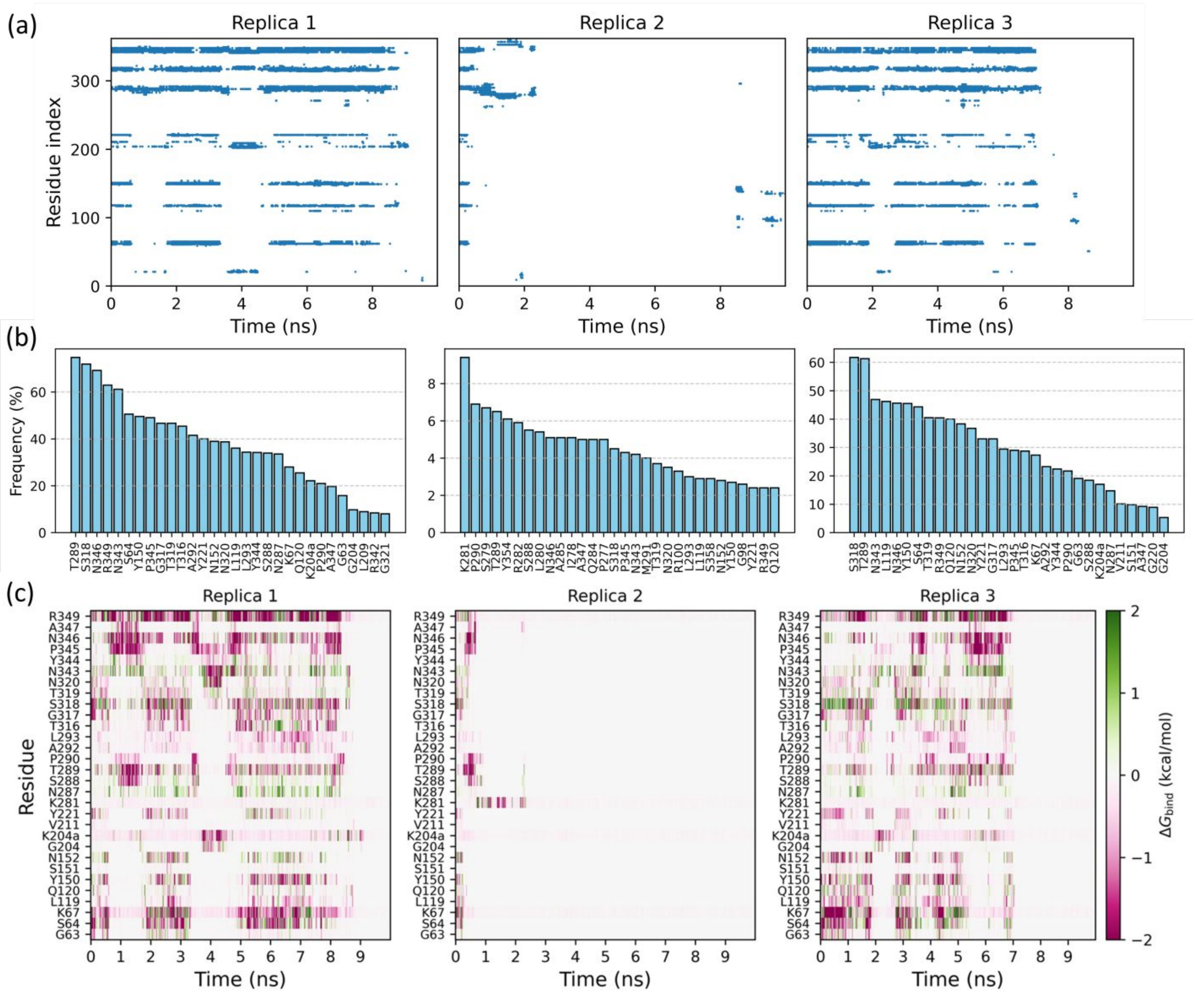

Figure S13. Contact of amino acid residues within 4.5 Å of LP06 during the unbinding process. (a) Time-dependent contact maps showing the amino acid residues in proximity to LP06 (< 4.5 Å) across three independent simulation replicas. Each dot represents a residue–LP06 contact at a specific time point. (b) Frequency histogram depicting the top 30 residues most frequently in contact with LP06 (< 4.5 Å) for each replica. (c) Time-resolved decomposition of binding free energy contributions (ΔG_{bind}) for the 30 most frequently contacting residues with LP06 across three replicas. Heatmaps display the per-residue contribution to the binding free energy (ΔG_{bind}) during the unbinding process. Rows correspond to the top 30 residues with the highest contact frequency, and columns represent simulation time. Color intensity indicates the magnitude of energy contributions, with magenta representing favorable interactions and green representing unfavorable interactions.

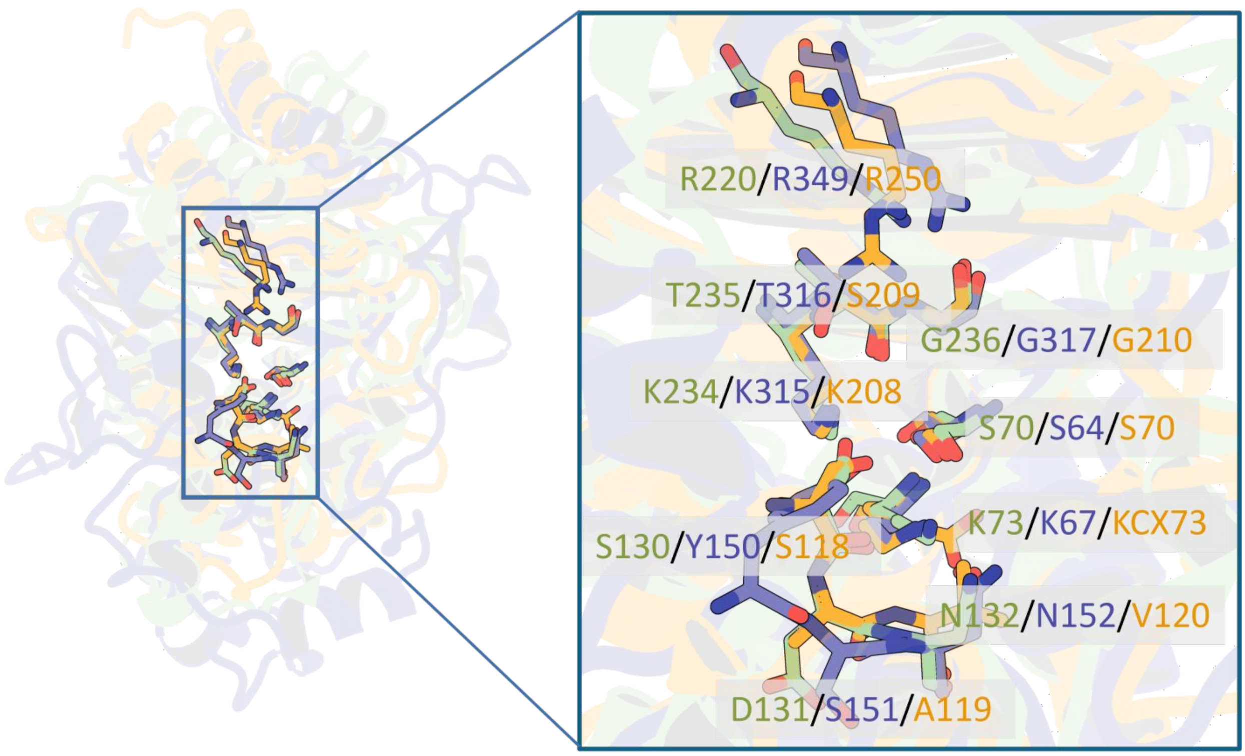

Figure S14. Superimposition of representative structures from Class A, C and D β-lactamases: KPC-2 (PDB ID: 5MGI) in green, PDC-3 (PDB ID: 8SDR) in violet, and OXA-24/40 (PDB ID: 5TG5) in orange. The conserved catalytic motifs and the the anchoring arginine are highlighted using stick representations. KCX73 in class D β-lactamases denotes the Nζ-carboxylated K73.

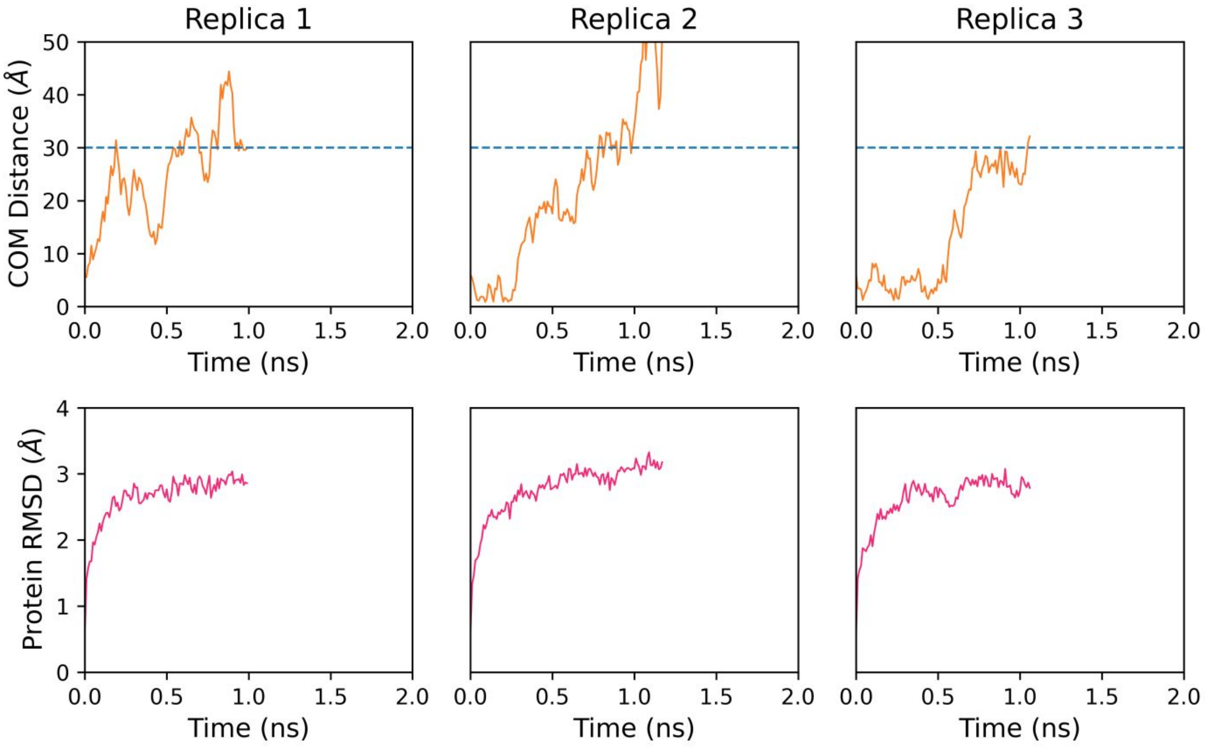

Figure S15. Time evolution of the ligand–protein center-of-mass (COM) distance (top) and protein Cα RMSD (bottom, referenced to the crystal structure) for three replicas at scaling factor λ = 0.2 in SMD. Unbinding occurs almost immediately (<1 ns) in all replicas, but the protein shows large, non-physical deviations (RMSD ≈ 3 Å), indicating overly aggressive scaling and a high risk of artifacts.

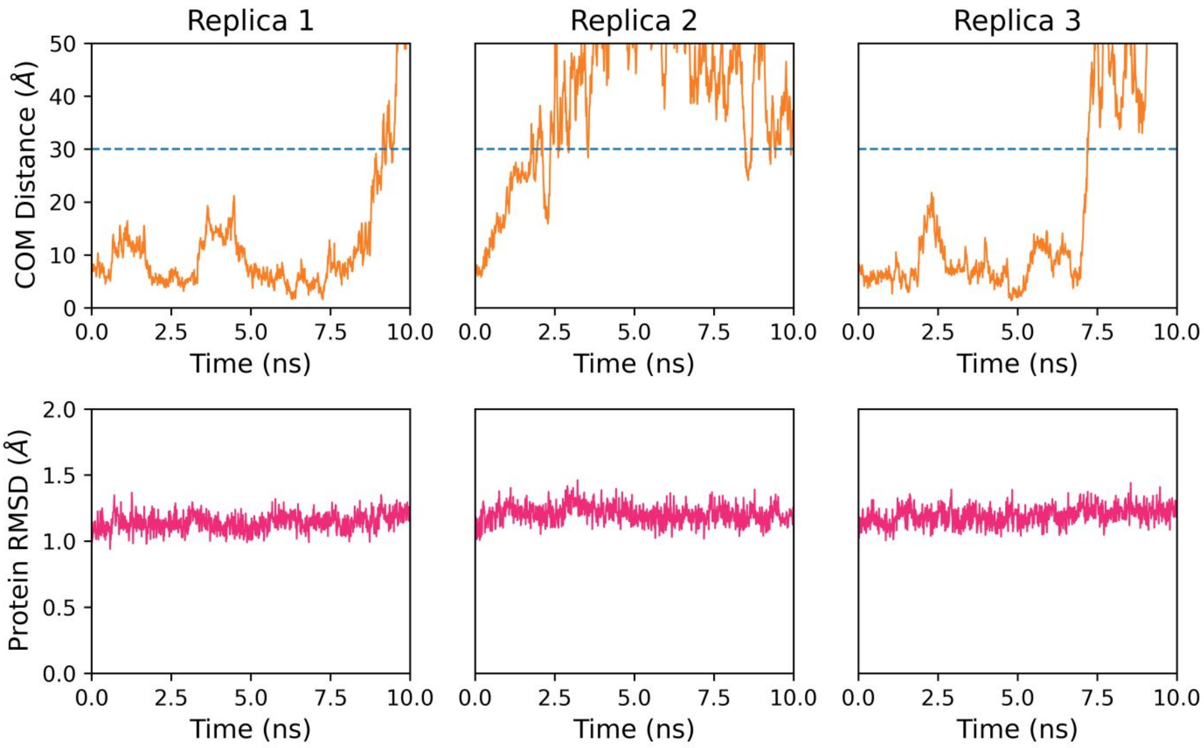

Figure S16. Time evolution of the ligand–protein center-of-mass (COM) distance (top) and protein Cα RMSD (bottom, referenced to the crystal structure) for three replicas at scaling factor λ = 0.4 in SMD. Unbinding is consistently achieved within ~10 ns, while the Cα trace remains compact and near-native (RMSD ≈ 1 Å), indicating that λ = 0.4 accelerates rare events while preserving the protein's conformation.

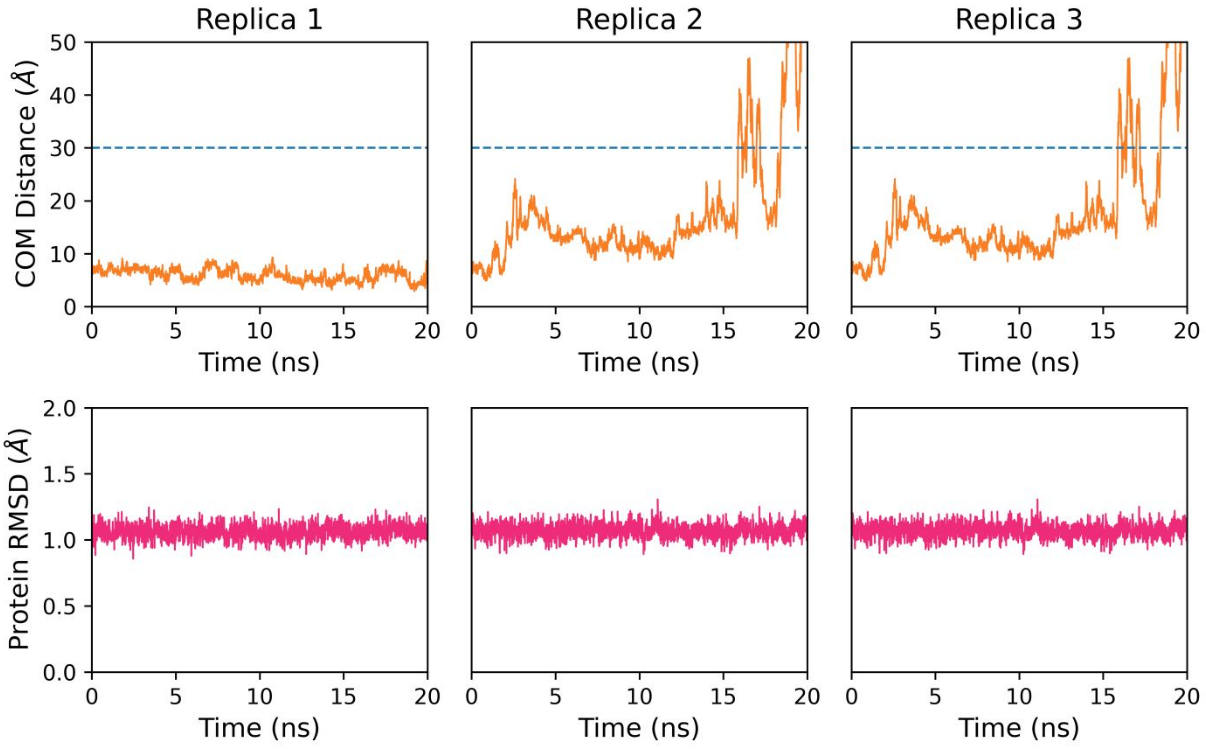

Figure S17. Time evolution of the ligand–protein center-of-mass (COM) distance (top) and protein Cα RMSD (bottom, referenced to the crystal structure) for three replicas at scaling factor λ=0.6 in SMD. Compared to λ = 0.4, unbinding proceeds more slowly (typically ~20 ns to cross the 30 Å COM threshold), and in Replica 1 the ligand even remains stably bound beyond 20 ns. Although protein conformation is preserved (RMSD ≈ 1 Å), λ = 0.6 provides only modest barrier reduction.
